# Metabolic regulation of the immune system in health and diseases: mechanisms and interventions

**DOI:** 10.1038/s41392-024-01954-6

**Published:** 2024-10-09

**Authors:** Tengyue Hu, Chang-Hai Liu, Min Lei, Qingmin Zeng, Li Li, Hong Tang, Nannan Zhang

**Affiliations:** 1 West China School of clinical medical, West China Second University Hospital, Sichuan University, Chengdu, China; 2 Center of Infectious Diseases, West China Hospital of Sichuan University, Chengdu, China; 3 Laboratory of Infectious and Liver Diseases, Institution of Infectious Diseases, West China Hospital, Sichuan University, Chengdu, China; 4 National Center for Birth Defect Monitoring, Key Laboratory of Birth Defects and Related Diseases of Women and Children, Ministry of Education, West China Second University Hospital, Sichuan University, Chengdu, China; 5 State Key Laboratory of Biotherapy/Collaborative Innovation Center of Biotherapy, West China Second University Hospital, Sichuan University, Chengdu, China; 6 Division of Renal and endocrinology, Qin Huang Hospital, Xi’an, China

**Keywords:** Immunological disorders, Immunopathogenesis, Adaptive immunity

## Abstract

Metabolism, including glycolysis, oxidative phosphorylation, fatty acid oxidation, and other metabolic pathways, impacts the phenotypes and functions of immune cells. The metabolic regulation of the immune system is important in the pathogenesis and progression of numerous diseases, such as cancers, autoimmune diseases and metabolic diseases. The concept of immunometabolism was introduced over a decade ago to elucidate the intricate interplay between metabolism and immunity. The definition of immunometabolism has expanded from chronic low-grade inflammation in metabolic diseases to metabolic reprogramming of immune cells in various diseases. With immunometabolism being proposed and developed, the metabolic regulation of the immune system can be gradually summarized and becomes more and more clearer. In the context of many diseases including cancer, autoimmune diseases, metabolic diseases, and many other disease, metabolic reprogramming occurs in immune cells inducing proinflammatory or anti-inflammatory effects. The phenotypic and functional changes of immune cells caused by metabolic regulation further affect and development of diseases. Based on experimental results, targeting cellular metabolism of immune cells becomes a promising therapy. In this review, we focus on immune cells to introduce their metabolic pathways and metabolic reprogramming, and summarize how these metabolic pathways affect immune effects in the context of diseases. We thoroughly explore targets and treatments based on immunometabolism in existing studies. The challenges of translating experimental results into clinical applications in the field of immunometabolism are also summarized. We believe that a better understanding of immune regulation in health and diseases will improve the management of most diseases.

## Introduction

The immune system removes pathogens and maintains balance in the body, with metabolism playing a crucial role in supporting immune functions.^1^ The interactions between metabolism and immunity have attracted many researchers. The relationship between metabolism and immunity have been considered bidirectional because of the prominent inflammatory responses in metabolic diseases and the variable metabolic pathways of immune cells.^2^ However, studies on the crosstalk between metabolic regulation and immune system is inseparable from the regulation of immune functions. This can be observed through changes in the phenotypes of immune cells, alterations in metabolic pathways and metabolic levels of immune cells, and changed cytokine levels. Therefore, we believe that the bidirectional relationship is essentially metabolic regulation of the immune system.

**Fig. 1:**
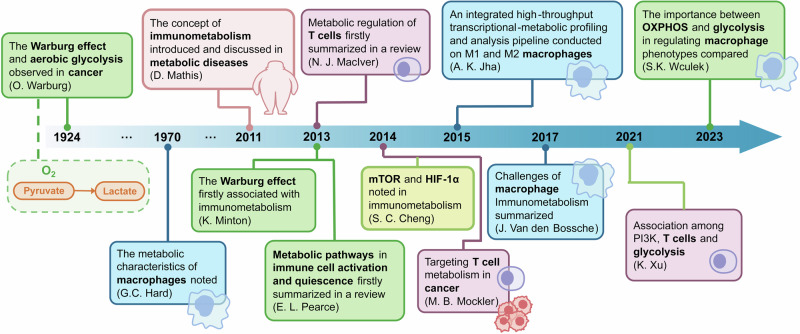
Timeline for metabolic regulation of the immune system. Events mainly involving new findings or important reviews on metabolic pathways are in green boxes. Events mainly focusing on macrophages and T cells are are in bule and purple boxes. Light green boxes show events involving important molecules in immunometabolism. A light red box shows a special event that the concept of immunometabolism has been introduced and discussed in metabolic diseases in 2011. Before 2011, the studies on Warburg effect in cancer and metabolic characteristics of macrophages both contributes to the development of the immunometabolism field. In the last more than twenty years, the concept immunometabolism has been generally accepted and studied. Abbreviation: OXPHOS oxidative phosphorylation, mTOR mechanistic target of rapamycin, HIF-1α hypoxia-inducible factor 1α, PI3K phosphatidyl-inositol 3 kinase

More than a decade ago, the concept of immunometabolism was proposed to summarize the interaction between metabolism and immunity (Fig. 1).^3^ In the beginning, researchers noted only inflammatory responses in metabolic disorders, including obesity, insulin resistance, and type 2 diabetes mellitus (T2DM), to define immunometabolism.^3–6^ After summarizing the differences of metabolic pathways in activated and quiescent immune cells, the definition of immunometabolism has been greatly expanded.^7,8^ Soon afterward, metabolic pathways of T cells have been discussed as a promising entry point for cancer immunotherapy.^9^ Then, benefiting from accessibility of measuring cellular metabolism of immune cells, immunometabolism has been introduced in studies on many other diseases, becoming an emerging and booming field. Metabolic regulation of the immune system becomes more and more clearer after the concept of immunometabolism being proposed.

Here, we are going to summarize metabolic regulation of the immune system in health and diseases by focusing on cellular metabolism and metabolic pathways in immune cells. First, metabolic pathways of immune cells, primarily including glycometabolism and lipid metabolism pathways, should be elaborated according to the phenotypes of immune cells. Second, we will discuss the changes in metabolic pathways of immune cells in different diseases. Although metabolic patterns of immune cells with proinflammatory or anti-inflammatory phenotypes can be highly generalized, studies in the context of different diseases can provide validation and identify specific characteristics or targets related to those diseases. Finally, our summary of studies on immunometabolism aims to further summarize existing and potential therapies targeting immunometabolism, ultimately improving the management of diseases in clinical practice.

## Metabolic pathways and reprogramming in immune cells

**Fig. 2:**
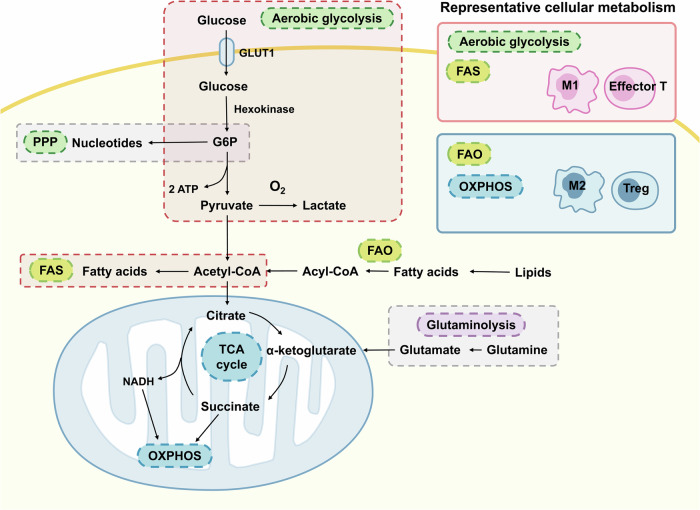
Metabolic pathways in immune cells. The most reported metabolic pathways in immune cells are glycolysis, especially aerobic glycolysis, fatty acid oxidation (FAO), fatty acid synthesis (FAS), and oxidative phosphorylation (OXPHOS). Aerobic glycolysis and FAS are always active in immune cells with proinflammatory phenotypes including M1 macrophages and effector T cells, while FAO and OXPHOS are always active in immune cells with anti-inflammatory phenotypes including M2 macrophages and regulatory T (Treg) cells. Pentose phosphate pathway (PPP) is a branch of glycometabolism but studies on immune cells are not enough. Glutamine is the most important amino acid in immunometabolism, of which the metabolism is associated with other pathways by α-ketoglutarate

The metabolism in immune cells is characterized by intricate links between metabolic reprogramming and immune cell activation.^10^ Metabolism contributes to immune cell activation and immune function, with immune cells adopting specific metabolic programs.^11^ Metabolic patterns of immune cells can be changed according to their different phenotypes, a process known as metabolic reprogramming.^12^ We are going to summarize the key metabolic pathways and metabolic reprogramming in immune cells (Table 1, Fig. 2). For the close association with energy production, the reprogramming of glycometabolism and lipid metabolism has gained much attention in immunometabolism. Amino acid signals have also shown their important roles in regulating immune responses.

**Table 1:** Metabolic pathways and metabolic characteristics of different immune cells

Immune cell	Glycolysis	FAO	OXPHOS	References
Macrophage	M1 macrophages mainly rely on aerobic glycolysis	M2 macrophages mainly rely on OXPHOS and FAO	M2 macrophages mainly rely on OXPHOS and FAO	^14,21^
T cell				
Naive T cell	In a quiescent state with low metabolic activity	A main energy source and default metabolic program	A main energy source and way of glucose metabolism	^73,88,91^
Effector T cell	With highly upregulated aerobic glycolysis, also known as the Warburg effect	Downregulated during T cell activation	Also upregulated, but less than aerobic glycolysis	^80,91^
Memory T cell	Not the default metabolic program; promoting rapid recall response of CD8^+^ memory T cells; depending on intracellular glycogen; main metabolic pathway for G6P	An energy source and the default metabolic program; with fatty acids as the preferent metabolites in the survival environment with limited glucose	An energy source and the default metabolic program	^73,76,80,84,88,91,95,97^
Treg cell	Not the default metabolic program	An energy source and the default metabolic program; with fatty acids as the preferent metabolites in the survival environment with limited glucose	An energy source and the default metabolic program	^76,80,84–88,91,95,97^
B cell				
Naive B cell	In a quiescent state with low metabolic activity	In a quiescent state with low metabolic activity	In a quiescent state with low metabolic activity	^113^
Activated B cell	Upregulated; B cells with chronic BAFF stimulation rapidly increase glycolysis; aerobic glycolysis not highlighted in nonneoplastic B cells; B1 cells have a higher rate of glycolysis than B2 cells	Germinal center B cells carry out active FAO	Upregulated; B1 cells have a higher rate of OXPHOS than B2 cells	^115,116,581^
Neutrophil	Mainly rely on glycolysis for ATP production	Unclear	Very limited because of low mitochondrial density	^134^
Dendritic cell	Upregulated during pathogen infection	Upregulated in activated dendritic cells	Upregulated in activated dendritic cells	^140,141^
Natural killer cell	Indispensable for the anti-tumor effect	Enhance the responses against infection and cancer	Cover the metabolic demand at a quiescent state	^149–152^
Group 2 innate lymphoid cell	Upregulated in the activated state	Supports the function during helminth infection	Always at steady state	^149,157,158^
Group 3 innate lymphoid cell	Upregulated in the activated state	Unclear	Upregulated in the activated state	^158,160^

*FAO* fatty acid oxidation, *OXPHOS* oxidative phosphorylation, *FAS* fatty acid synthesis, *G6P* glucose-6-phosphate

### Metabolic pathways and reprogramming in macrophages

Macrophages are a heterogeneous population of immune cells with different functions. Diverse macrophages have been simply categorized into two polarizations: M1 and M2 subsets, simplifying the complexity in related studies.^13^ M1 macrophages are proinflammatory and are activated through a classical activation by interferon-γ (IFN-γ), interleukin (IL)-1, and LPS.^14^ M2 macrophages are anti-inflammatory and are activated through an alternative pathway by IL-4 and IL-1.^14^ M2 macrophages can be further subdivided into M2a, M2b, M2c, and M2d macrophages according to the different stimuli.^15–18^ How to polarize macrophages through adding certain stimuli in vitro has been described.^19^ In specific diseases, certain macrophage phenotypes have been identified.^20^ The metabolic reprogramming that occurs during the phenotypic and functional changes of macrophages is a highly concerned and somewhat controversial topic in immunometabolism.

#### Glycometabolism in macrophages

Proinflammatory M1 macrophages have higher glucose consumption and lactate release than M2 macrophages, mainly relying on aerobic glycolysis, while anti-inflammatory M2 macrophages mainly rely on oxidative phosphorylation (OXPHOS).^14,21^ With the immunometabolism rapidly developing, the mechanisms of macrophage activation and phenotypic changes are becoming clearer. However, contradictory results are also emerging. Directly relating glycolysis to proinflammatory effects or oxidative metabolism to anti-inflammatory effects is not able to summarize the full picture of immunometabolism.^22^

Aerobic glycolysis, also known as the Warburg effect, is a process in which pyruvate is converted into lactate instead of being oxidized in the mitochondria to produce 2 molecules of ATP under aerobic conditions.^23^ The Warburg effect was first reported in cancer by Warburg et al. one hundred years ago and has since received increasing attention in the field of cancer metabolism.^24–27^ Then, the Warburg effect has gradually been shown to play roles in other diseases.^28,29^ Nearly a decade ago, the Warburg effect has been linked to immunometabolism in discussions on how aerobic glycolysis conducts post-transcriptional control in T cell function.^30,31^ After observing significantly increased glycolysis in multiple activated immune cells under aerobic or anaerobic conditions, aerobic glycolysis has been recognized as an important metabolic pathway in the field of immunometabolism.^32,33^

In M1 macrophages, glycolysis is a crucial metabolic event with many factors involved.^21^ Downregulation of glycolysis in macrophages is always accompanied by reduced secretion of inflammatory cytokines.^34^ Hypoxia-inducible factor (HIF) 1α is a well-studied molecule that induces glycolysis and M1 polarization. The overexpression of HIF-1α can upregulate glycolysis and pentose phosphate pathway (PPP) of macrophages.^35^ Accumulated succinate and citrate from the altered tricarboxylic acid (TCA) cycle, mechanistic target of rapamycin (mTOR) complex 1 (mTORC1), and pyruvate kinase muscle isozyme M2 (PKM2) have been identified as upstream regulators of HIF-1α during glycolysis regulation and macrophage polarization.^36–41^ However, binding of succinate to activate succinate receptor on macrophages can sometimes promote immunosuppressive macrophage polarization through the HIF-1α signaling pathway.^42^ Citrate can increase proinflammatory factors and is accumulated at high concentrations outside M1 macrophages but not M2 macrophages.^43,44^ Although mTORC1 has been considered a key regulator of glycolysis, its role in regulating macrophage functions remains unclear.^45^ After knocking down mTORC1 in macrophages, an unexpected enhancement in the function of M1 macrophages has been observed, along with impaired glycolysis.^45^ Enhanced PKM2-dependent glycolysis has been observed simultaneously with upregulated M1 polarization.^46,47^ The upregulated PPP of M1 macrophages has received less attention compared to glycolysis, although some previous reviews have discussed the PPP of macrophages.^48,49^ Upregulation of the PPP in macrophages can also enhance inflammatory responses and contribute to the development of inflammatory diseases.^50,51^

Glycometabolism in M2 macrophages is partly controversial, due to the role of glycolysis.^21^ As mentioned before, it has been widely accepted that OXPHOS is associated anti-inflammatory macrophages, while glycolysis is associated with proinflammatory macrophages. However, the metabolic pathways that can distinguish macrophages with different phenotypes are likely to be OXPHOS instead of glycolysis.^52^ For M2 polarization, OXPHOS, but not glycolysis, is necessary.^52^ Some researchers have reported that inhibiting glycolysis in unpolarized macrophages cannot significantly affect M2 differentiation.^53^ In addition, inhibiting glycolysis in M2 macrophages can prevent M1 polarization.^54^ Thus, the question of whether glycolysis is active or inactive in M2 macrophages remains unclear.

#### Lipid metabolism in macrophages

In addition to OXPHOS, active fatty acid oxidation (FAO) is another metabolic characteristic of M2 macrophages.^55^ Fatty acids serve as precursors for producing inflammatory mediators, so catabolism including FAO is considered anti-inflammation by some researchers.^56^ Peroxisome proliferator-activated receptor (PPAR) is an important promotion factor for FAO of macrophages, downstream of receptor-interacting protein kinase 3 (RIPK3).^57^ Activation of PPAR induces M2 polarization.^57^ However, some researchers have found that inhibiting FAO does not disrupt M2 polarization, indicating the complex roles of FAO in regulating macrophage functions.^58^ FAO can also induce inflammasome activation in M1 macrophages, further indicating that associating FAO with anti-inflammation cannot summarize the entire story.^22,59^

#### Amino acid metabolism in macrophages

Amino acid metabolism in macrophages needs to be studied. Glutamine catabolism of macrophages can induce M2 polarization, and glutamine is essential for M2 polarization.^60–63^ Through glutaminolysis, α-ketoglutarate can be produced, inducing FAO and epigenetic reprogramming to promote M2 macrophage polarization.^64^ In environments with low levels of glutamine, macrophages can upregulate glutamine synthetase activity to secrete glutamine, but how the glutamine synthesis capability affects the functions of macrophages is still unclear.^65^ Serine synthesis in M1 macrophages supports IL-1β production through phosphoglycerate dehydrogenase signaling, and also activates NACHT, LRR, and PYD domains-containing protein 3 (NLRP3) inflammasome.^66^ Serine metabolism has been also reported to regulate macrophage polarization. Inhibiting serine synthesis can induce M1 polarization and reduce M2 polarization by activating the JAK/STAT signaling pathway.^67^ However, when serine synthesis induces M1 polarization, the inflammasome is not activated by serine metabolism.^68^ Upregulating serine synthesis can induce M2 polarization.^69^

Lipid synthesis might be the other side of lipid catabolism, which is positively associated with inflammation. Inhibiting lipid synthesis and enhancing lipid catabolism can synergistically promote M2 polarization and alleviate inflammation.^70^ Inhibiting lipid synthesis of macrophages can reduce the levels of proinflammatory cytokines.^71^ Lipid synthesis also impacts phagocytosis of macrophages.^72^ The sterol responsive element binding protein (SREBP)-1a-dependent lipid synthesis regulated by mTORC1 is essential for phagocytosis in macrophages.^72^

### Metabolic pathways and reprogramming in T cells

The metabolic activity of T cells depends on their phenotypes and subtypes. Naive T cells remain quiescent with limited requirements for inducing metabolic pathways.^73^ As naive T cells differentiate into effector T cells, there is an increased requirement for biomass and the rapid proliferation, leading to active metabolism.^74^ The metabolic changes in T cells to meet the bioenergetic demand for rapid proliferation are also defined as metabolic reprogramming.^75^ As an immunosuppressive subset, regulatory T (Treg) cells exhibit distinct metabolic features compared to naive T cells and effector T cells.^76^ The metabolic features of memory T cells differ from those of other T cells.^73^ We will attempt to summarize the metabolic features of T cells through different metabolic pathways.

#### Glycometabolism in T cells

Naive T cells are regarded as a quiescent cell population with low metabolic demands.^77^ For only requiring energy for survival and migration, naive T cells utilize OXPHOS as the main metabolic pathway to metabolize glucose.^78,79^ Through OXPHOS in the mitochondria, naive T cells metabolize pyruvate to access an optimal yield of adenosine triphosphate (ATP) per glucose molecule.^74^

During and after T cell activation, the glucose uptake significantly increases and aerobic glycolysis occurs in T cells.^74^ To increase the glucose uptake, the expression of glucose transporter (GLUT) proteins is upregulated in T cells.^80^ Overexpression of GLUT3 can enhance glucose uptake in CD8^+^ T cells.^81^ GLUT1 can be upregulated by the phosphatidylinositol 3-kinase (PI3K)/ protein kinase B (Akt) pathway, which can be activated by CD28 co-stimulation with T-cell receptor (TCR) signals, and then participates in activating CD4^+^ T cells.^80,82,83^ The reason effector T cells significantly upregulate aerobic glycolysis might be to provide glycolytic precursors for biosynthetic reactions.^73^ An important factor for aerobic glycolysis, HIF-1α, is highly expressed in effector T cells under aerobic conditions.^80^ HIF-1α can enhance aerobic glycolysis by inducing the transcription of the enzymes pyruvate dehydrogenase kinase 1 (PDK1) and lactate dehydrogenase A (LDHA). Although OXPHOS is also increased during T cell activation, the extent is lower compared to aerobic glycolysis.^80^

Memory T cells and Treg cells also exhibit distinct metabolic characteristics. Memory T cells and Treg cells mainly rely on OXPHOS for more efficient energy sources like naive T cells.^76^ The metabolic pathways regulating glycolytic rate in memory T cells have been more fully studied than naive T cells. Memory T cells with high metabolic fitness are prepared to engage in active metabolism on demand, aiming for a rapid immune response.^73^ In the rapid recall response of CD8^+^ memory T cells, glycolysis plays a promoting role with intracellular glycogen serving as the major carbon source instead of extracellular glucose.^84^ After antigenic stimulation, TCR signaling phosphorylates glycogen phosphorylase together with LCK and ZAP70, then inducing glycogenolysis and increasing glucose-6-phosphate (G6P). Glycolysis is the main downstream metabolic pathway for G6P, and PPP also metabolizes G6P. Thus, memory CD8^+^ T cells rely specifically on glycogen for activation. However, studies on metabolic characteristics of memory CD4^+^ T cells are still lacking. The role of glucose metabolism in Treg cells is a controversial issue, with significantly varying results across different studies.^84^ In conjunction with the interaction between Treg cells and other T cell subsets, the metabolic network in Treg cells becomes increasingly complex. Treg cells and IL-17-producing T helper (Th17) cells cells are closely connected in existing studies and mutually antagonistic both in differentiation and function.^85^ Th17 cells have proinflammatory effects, while Treg cells have immunosuppressive functions. As a subset of effector CD4^+^ T cells, Th17 cells exhibit active glycolysis and utilize the pentose phosphate pathway. Treg cells depend more on FAO and OXPHOS for energy.^86^ Decreasing glucose levels results in reduced differentiation of Th17 cells but enhances the differentiation of Treg cells, thereby enhancing their immunosuppressive activity.^85,87^

#### Lipid metabolism in T cells

In addition to metabolizing glucose-derived pyruvate through OXPHOS, naive T cells carry out FAO as the default metabolic program, which is also an efficient way to generate energy.^74,88^

Increased anabolism is a characteristic of the metabolic reprogramming during and after activation of T cells.^74^ FAS is induced as a key cellular lipid biosynthetic pathway for T cell activation, accompanied by the downregulation of FAO. mTOR is a serine/threonine kinase regulating cellular metabolism, including T cell metabolism.^89^ T cells depend on mTOR to meet the demands for nutrient uptake during activation and differentiation.^90^ The upstream molecules of mTOR in immune signal pathways are numerous. TCR, co-stimulatory receptors, and cytokines can activate mTOR.^90^ Upon activation of mTORC1, both glycolysis and de novo FAS are enhanced in T cells.^91^ SREBPs are important downstream molecules of mTORC1 that regulate the metabolism of fatty acids and cholesterol.^80^ In effector CD8^+^ T cells, SREBPs induce the expression of the rate limiting enzymes in FAS and cholesterol synthesis, such as acetyl-CoA carboxylase (ACC).^74,91^ Additionally, PPARγ serves as a critical downstream molecule of mTORC1 in regulating fatty acid uptake in effector CD4^+^ T cells.^92^ PPARγ can directly bind to genes involved in fatty acid metabolism, including ACC1, thereby promoting fatty acid uptake in CD4^+^ T cells.^75^ The de novo lipid synthesis mediated by ACC1 plays a critical role in the differentiation of Th17 cells from CD4^+^ T cells.^88^ The upstream or downstream relationships between PPARγ and SREBPs in T cell lipid metabolism have not been reported.^93,94^

Like naive T cells, memory T cells and Treg cells maintain FAO and OXPHOS as the default metabolic program.^80,88,91,95^ Fatty acids, rather than glucose, are the preferent metabolites for memory T cells and Treg cells due to the survival environment with limited glucose.^95^ The survival of certain subsets of memory CD8^+^ T cells is dependent on the uptake and metabolism of exogenous lipids.^96^ Different from effector CD8^+^ T cells, the development of memory CD8^+^ T cells does not depend on SREBP activity and can be inhibited by mTOR activation.^97^ The features of lipid metabolism in memory CD4^+^ T cells remain unclear. Treg cells also prefer to uptake extracellular fatty acids rather than undergo de novo FAS.^88^ Forkhead/winged helix transcriptional factor P3 (FoxP3) is a nuclear-specific transcription factor expressed in Treg cells, important for maintaining immunological self-tolerance.^98^ Foxp3 enables Treg cells to tolerate high fatty acid concentrations by inducing FAO and triglyceride synthesis.^76^ Cytotoxic T-lymphocyte-associated antigen 4 (CTLA-4) and programmed death 1 (PD-1) can act as upstream molecules to upregulate Foxp3 expression.^99^

#### Amino acid metabolism in T cells

Amino acid metabolism in T cells is complex, with studies focusing on amino acid metabolism during T cell activation. Multiple amino acids are necessary for the activation, proliferation and function of T cells.^100^ Alanine is involved in protein synthesis rather than catabolism during T cell activation. While alanine can be synthesized from pyruvate through transamination, extracellular alanine is still required for naive T cell activation and memory T cell restimulation.^101^ Arginine concentration decreases in activated T cells, while the levels of other amino acids remain stable or increase.^102^ Increasing L-arginine levels in the experiment, can lead to a shift from glycolysis to OXPHOS, promoting the formation of memory T cell.^102^ Also, the uptake of cysteine and cystine is necessary for T cell activation, but it is noteworthy that cysteine can modify electrophilic compounds to impair T cell activation.^100,103^ Glutamine has been recognized as an immunomodulatory nutrient for a long time. The activation of naive T cells is accompanied by rapid glutamine uptake, depending on the amino acid transporter ASCT2, which could induce Th1 and Th17 cells activation in immunity and autoimmunity.^104^ Leucine is considered as an important nutrient signal for activating mTORC1.^105^ Methionine has been identified as a key nutrient signal in epigenetic reprogramming in CD4^+^ Th cells, including Th17.^106^ Serine controls proliferative capacity of effector T cells by supplying glycine and one-carbon units for de novo nucleotide biosynthesis.^107^ In summary, although studies on the synthesis and catabolism of amino acid in T cells are lacking, the results on how amino acid uptake regulates T cells already indicate the important role of amino acid in connecting metabolism and T cells.

### Metabolic pathways and reprogramming in B cells

B cells are lymphocytes that develop from hematopoietic precursor cells and maturate in an ordered and selective process.^108^ Several B cell subsets have been identified. B1 cells, including B1a and B1b, are mainly generated in the foetal liver and play important roles in innate immunity.^109^ B2 cells, consisting of follicular B and marginal zone B cells, are derived from the bone marrow and play conventional roles in adaptive immunity.^109^ Regulatory B (Breg) cells are immunosuppressive cells that support immunological tolerance and can differentiate from immature B cells, mature B cells, and plasmablasts.^110^

B cells at different stages of maturity vary in metabolic activity, and the metabolic pathways in different B cell subsets are also different.^111^ Like T cells, resting B cells have a limited requirement for metabolic pathway activation, while activated B cells undergo metabolic reprogramming to meet the bioenergetic demands for rapid proliferation and antibody production.^112,113^ However, studies on metabolic pathways in B cells are significantly less than that in T cells.

#### Glycometabolism in B cells

After B cell activation, glucose uptake increases dramatically.^114^ The expression of GLUT1 and mitochondrial mass in B cells are upregulated following stimulation with lipopolysaccharide (LPS) or B cell receptor to enhance glucose uptake.^115^ Unlike the metabolic reprogramming in T cells, subsequently increased glycolysis and OXPHOS in B cells depends on tolerance.^115^ Anergic B cells, which do not respond to antigen, remain metabolically quiescent, while B cells stimulated by chronic B cell-activating factor rapidly increase glycolysis.^115,116^ In addition, aerobic glycolysis is not highlighted in nonneoplastic B cells, leading researchers to consider glycometabolic patterns in B cells as conventional.^117,118^

#### Lipid metabolism in B cells

Although B cells have metabolic requirements for lipids, studies on lipid metabolism in B cells are limited. As mentioned before, FAS is an important type of metabolism for T cell activation, with corresponding downregulation of FAO. However, germinal center B cells, critical cells for long-term humoral immunity, conduct active FAO and minimal glycolysis to achieve rapid proliferation, indicating different metabolic patterns from T cells.^119^ The important roles of short-chain fatty acids in regulating immune functions in B cells have been reported, also indicating that lipid metabolism in B cells requires more attention.^120,121^

#### Amino acid metabolism in B cells

Due to the ability of B cells to secrete large amounts of antibodies, amino acid metabolism and related signaling are particularly important. Glutamine is the most frequently reported amino acid for B cells. The uptake and utilization of extracellular glutamine are important for B cell activation.^122^ Glutamine can be catabolized to generate energy for B cells through a glucose-independent TCA cycle, which promotes cell proliferation under hypoxia and glucose deficiency.^123^ Glutamine metabolism can also mediate mitochondrial function enhancement.^124^ Furthermore, glutamine can promote the generation of Breg cells in different subsets of B cells through the mTOR/glycogen synthase kinase 3 pathway.^125^ The number of IgA^+^ plasma cells in the ileum can be increased by glutamine supplementation.^126^ Other amino acids are also important for B cells. Arginine undergoes increased methylation after B-cell activation, which is essential for the proliferation, differentiation, and survival of B cells.^127,128^ Tryptophan can be catabolized through a pathway mediated by the indoleamine 2,3-dioxygenases, whose members negatively regulate B cell proliferation.^129^ Leucine nutrient preferring B cells induce immune escape from tumors.^130^

### Metabolic pathways and reprogramming in neutrophils

Neutrophils are short-lived innate immune cells that regulate acute injury and repair, cancer, autoimmunity, and inflammatory processes.^131,132^ Neutrophils can act as the first responders to acute inflammation, contributing to anti-inflammatory effects, and also play important roles in chronic inflammation.^133^ The existence of different neutrophil subsets has been proven, but studies often do not focus on specific subsets.^134^ Neutrophils mainly rely on glycolysis for ATP production with very limited OXPHOS because of their low mitochondrial density.^135^ In environments where glucose availability is limited, FAO is an alternative pathway for obtaining energy.^134^

### Metabolic pathways and reprogramming in dendritic cells

Dendritic cells are innate immune and antigen-presenting cells that communicate environmental signals with T cells to bridge innate and adaptive immunity.^136^ Dendritic cells have complex subsets and participate in protective proinflammatory responses and tolerogenic immune responses.^137,138^ Dendritic cells undergo rapid metabolic reprogramming during the generation of specific immune responses, but the cellular metabolism of dendritic cells is still unclear.^139^ During pathogen infection and migration toward lymph nodes, metabolic reprogramming toward glycolysis in dendritic cells, which can be induced by the chemokine-mediated HIF-1α activation.^140,141^ In some other cases, upregulated FAO and OXPHOS have also been observed in activated dendritic cells, and differences in metabolic reprogramming can be caused by different Toll-like receptors.^141^ FAS is indispensable for the development and activation of dendritic cells.^142,143^ Lipid accumulation in dendritic cells can impair their capacity to process antigens.^144^

### Metabolic pathways and reprogramming in other innate immune cells

Innate lymphoid cells (ILCs), including natural killer (NK) cells, non-cytotoxic ILC1s, ILC2s and ILC3s, are emerging innate immune cells that participate in immune responses to pathogens.^145–147^ NK cells are important cells with antiviral and anti-tumor effects.^148^ OXPHOS can cover the metabolic demand of NK cells at a quiescent state.^149^ To maintain their functions including anti-tumor effect, glycolysis has been proven to be indispensable.^150,151^ FAO can enhance the responses of NK cells against infection and cancer.^152^ FAS has also been found to be required for proinflammatory effect of NK cells.^153^ Because their function of exerting direct cytotoxic responses is similar to CD8^+^ T cells, studies on the metabolic patterns of NK cells may be inspired by those on CD8^+^ T cells.^154^ Studies on the cellular metabolism of non-cytotoxic ILC1s, ILC2s and ILC3s are more limited than that on NK cells.^155^ The transcriptional and epigenetic identity of ILCs in the small intestinal has been reported, which may be helpful to study their cellular metabolism.^156^ It has been noted that FAO predominantly supports the function of ILC2s during helminth infection.^149,157^ In activated ILC2s, glycolysis is upregulated and OXPHOS is at steady state.^158^ In activated ILC3s, both glycolysis and OXPHOS are upregulated.^159,160^

## Metabolic regulation of the immune system in diseases

### Immunometabolism in cancers

**Fig. 3:**
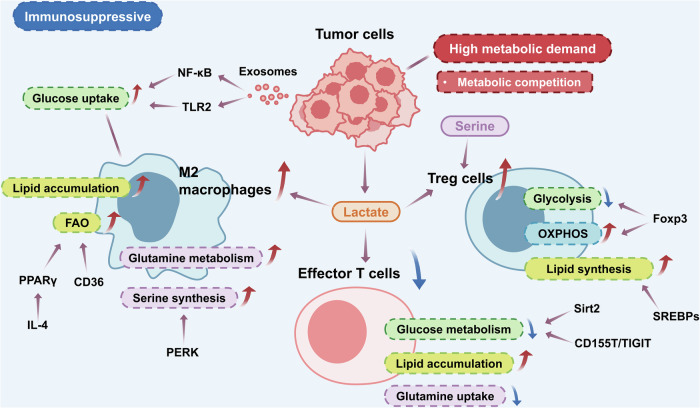
Metabolic regulation of immune cells in cancer. During the development and progression of cancer, dramatic metabolic reprogramming happens in tumor cells and immune cells. Tumor cells have high metabolic demand and metabolic competition with immune cells. The tumor microenvironment is an immunosuppressive environment with the proportion of proinflammatory immune cells decreasing and anti-inflammatory cells increasing. Tumor derived-lactate is an important metabolite to regulate the phenotypes of immune cells. M2 macrophages have an abnormally high capacity to take up glucose, while glucose metabolism is reduced in effector T cells and glycolysis. Nuclear factor-kappaB (NF-κB), Toll-like receptor-2 (TLR2) and forkhead/winged helix transcriptional factor P3 (FoxP3) can regulate glucose metabolism. Lipid accumulation is upregulated in M2 macrophages and effector T cells, and lipid synthesis is upregulated in regulatory T (Treg) cells mediated by sterol regulatory-element binding proteins (SREBPs). Fatty acid oxidation (FAO) is active in M2 macrophages, with peroxisome proliferator-activated receptor γ (PPARγ) and CD36 involved in the regulation. Glutamine metabolism and serine synthesis is increased in M2 macrophages, and protein kinase RNA-like ER kinase can upregulate the serine synthesis. In effector T cells, glutamine uptake is reduced

During the development and progression of cancer, dramatic metabolic reprogramming occurs in many cells, which has been fully discussed in studies focused on cancer metabolism.^161–163^ Immune cells also change their metabolic patterns to adapt to the stressful microenvironment of hypoxia and nutrient deprivation, simultaneously causing changes in immune function (Fig. 3).^164^ In addition to metabolic pathways, metabolites in the tumor microenvironment (TME) have also helped researchers to understand metabolic regulation of immune cells in cancer.^165^

#### Glycometabolism of immune cells in cancer

The most recognized metabolic phenotype of cancer cells is the altered glucose metabolism, which is known as Warburg effect or aerobic glycolysis.^166,167^ Similar to some immune cells, including effector T cells, tumor cells, tumor cells prefer to catalyze glucose into lactate instead of carbon dioxide, even in an oxygen-sufficient environment.^168^ Which should be noted is that in a variety of cancers, immune cells are a component of the TME, and their metabolic pattern is a topic different from that of tumor cells.^169^ The TME is an immunosuppressive environment in which glucose metabolism is altered in immune cells, which participate in building the environment.^79^

The metabolism of macrophages has been shown to be altered in the TME, and then change the polarization and anti-tumor response of macrophages.^170^ Tumor-associated macrophages cannot be completely classified into the M1 and M2 subtypes because of their general M2 phenotype, which promotes tumor growth and invasion.^171^ Polarization to the immunosuppressive phenotype has been proven to be caused by tumor-derived exosomes, which induce glycolytic-dominant metabolic reprogramming.^172^ Through Toll-like receptor-2 and nuclear factor-kappa B (NF-κB), tumor-derived exosomes can increase glucose uptake of macrophages. M2-like tumor-associated macrophages have an abnormally high capacity to take up intratumoral glucose, which has been proven to induce the hexosamine biosynthetic pathway and cardiac O-GlcNAcylation, subsequently promoting metastasis and chemoresistance.^173^

In a TME with low glucose and high lactate levels, the metabolic phenotypes of T cells change to promote immune tolerance. Foxp3, a Treg transcription factor, has been considered as a potential factor that regulates the metabolism of T cells in the TME. Foxp3 can suppress myelocytomatosis oncogene (Myc) and glycolysis, enhance OXPHOS, and increase nicotinamide adenine dinucleotide oxidation, possibly resulting in a metabolic advantage of Treg cells in the TME.^79^ In colorectal cancer, glucose intake and glycolysis are highly activated in Treg cells. Treg-specific MondoA knockout can induce Th17-like Treg cells to promote the initiation of cancer, indicating the important role of the MondoA-TXNIP axis in regulating the metabolism of Treg cells.^174^ The glucose metabolism of effector T cells in cancer is always reduced. In advanced non-small-cell lung cancer, upregulation of Sirtuin 2, which can suppress glycolysis in T cells, is associated with negative response to immunotherapy.^175^ In sarcoma, glycolysis of T cells is suppressed by the low glucose in the TME, and then mTOR activity and IFN-γ production are decreased, promoting tumor progression.^163^ In gastric cancer, glucose intake of CD8^+^ T cells is suppressed by cancer cells through CD155T/TIGIT signaling and the effector function is impaired.^176^

In contrast to studies on macrophages and T cells, studies on immunometabolism have focused on the malignant phenotype of B cells instead of considering them to be components of the TME. B-cell malignancies, including various leukemias and lymphomas, are prevalent worldwide.^177,178^ The metabolism of malignant B cells is complex and has been fully discussed in previous reviews.^179–181^ As cells with a low mitochondrial number and a small cytoplasmic volume, B cells are limited in obtaining glucose.^182^ However, oncogenes can drive B cells to obtain additional glucose, even resulting in permanently increased metabolic demands. Compared with that in healthy cells, the importance of pyruvate, the key product of glycolysis, is lowered for mitochondrial metabolism in malignant B cells, while glutaminolysis becomes more important.^179,183^ Flux of the PPP has been observed in chronic lymphocytic leukemia, where it protects malignant B cells from oxidative stress.^183^

#### Lipid metabolism of immune cells in cancer

Lipid metabolism of immune cells has been reported to be changed in cancer, participating in building an immunosuppressive microenvironment and tumor progression.

The intracellular metabolic lipid profiles of macrophages undergo significant changes in the TME, partly contributing to their pro-tumor effects.^184^ Generally, lipid accumulation and metabolism and M2 polarization are enhanced in macrophages when they participate in tumorigenesis or tumor progression.^185^ In lymphoma and myeloma mice, highly expressed CD36 induces macrophages to accumulate lipids and upregulate FAO to obtain energy.^185^ In breast cancer, M2 macrophages is associated with poor survival.^186^ Researchers have defined specific macrophage subsets with highly expressed genes related to lipid metabolism including fatty acid binding protein (FABP) 3, FABP4, FABP5, and myeloid cells 2, to provide a novel direction for combating breast cancer and anti-lung metastasis.^187^ PPARγ is considered as an important factor for FAO upregulation to induce M2 polarization in a breast cancer model.^188^ PPARγ can be induced by S100A4, which is highly abundant in macrophages and can be induced by IL-4. Some researchers even believe that long-chain fatty acid metabolism can control the M2 phenotype of macrophages in cancer, and lipid droplets are essential.^189^ However, we believe that although the polarization is important for elaborating the roles of macrophages in disease states, how lipid metabolism in macrophages promotes cancer progression cannot be completely generalized by abnormal polarization due to the complexity of the underlying mechanisms in addition to polarization. In prostate cancer, a special group of macrophages has shown a high lipid accumulation depending on the scavenger receptor Marco which can be activated by IL-1β.^190^ Macrophages with a high lipid accumulation can release CCL6 to promote cancer cell migration.

In the TME with a relative absence of glucose, T cells tend to accumulate lipids for obtaining energy.^88^ However, abnormal lipid accumulation in the TME can change the normal lipid accumulation and metabolism of T cells, causing dysfunction of effector T cells.^191^ Increased concentrations of lipids have been observed in CD8^+^ T cells of murine colon cancer and melanoma, inducing lipid peroxidation and activating p38 kinase, promoting CD8^+^ T cell dysfunction in the tumors.^192^ During the metabolic dysregulation, the expression of the scavenger receptor CD36 is increased its expression on CD8^+^ T cells, suggesting that this receptor is a potential immunometabolic target. Activated p38 mitogen-activated protein kinases (MAPKs) and lipid oxidation pathways have also been reported in the T cells from male breast cancer patients.^193^ However, as the central to Treg cell activation, the upregulation of lipid metabolism in Treg cells can drive Treg cells to enhance immunosuppression in the TME.^194^ Intratumoral Treg cells exhibit increased SREBP activity, indicating the upregulation of fatty acid synthase.^195^ Inhibiting lipid synthesis and metabolic signaling via SREBPs can enhance anti-tumour immune responses, which are related to PD-1.^195^

There are only a few published studies on lipid metabolism in B cells in cancer, and studies on lipid metabolism of healthy B cells have been summarized in a previous review.^196^ Although the evidence is limited, lipid metabolism of B cells in cancer has been considered to be altered. In chronic lymphocytic leukemia, changes of lipid metabolism in malignant B cells have been reported.^197,198^ Lipases and phospholipases are significantly overexpressed in chronic lymphocytic leukemia cells, and a lipase inhibitor can induce apoptosis of B-cells.^198^ PPARδ can change cholesterol metabolism and related signaling in malignant B cells.^197^ More than ten years ago, PPARα has been discovered to be an effective therapeutic target for murine B-cell lymphoma through the regulation of lipid metabolism.^199^

In other immune cells, abnormal lipid accumulation has also been reported in cancer. Lipid accumulation in dendritic cells is associated with the decreased antigen processing ability, which has been proven in ovarian cancer and hepatocellular carcinoma (HCC).^200^ Although NK cells are important in tumor immunotherapy, how their metabolism changes in cancer is unclear. In murine colorectal carcinoma and melanoma, surgery can increase the lipid accumulation in NK cells, by upregulating MSR1, CD36 and CD68 and decreasing their ability to lyse tumor cells.^201^ In HCC, hepatocytes can produce excessive cholesterol and enhance lipid accumulation in NK cells, resulting in deficient NK cell cytotoxicity.^202^ In aggressive B-cell lymphoma, the high levels of fatty acids in the environment can impair the function of NK cells.^203^

#### Amino acid metabolism of immune cells in cancer

Amino acids are metabolic regulators that support cancer cell proliferation, and also essential regulators of immune cells.^204,205^ However, existing studies have considered amino acids to be extrinsic signaling molecules through which immune cells to regulate immune responses in cancer. The amino acid anabolism and catabolism of immune cells in cancer are still difficult to summarize, but we believe that amino acid metabolism of immune cells in cancer has certain unique characteristics based on limited evidence.

The expression of EPHB2, which is an important gene of glutamine metabolism, is high in macrophages, especially M2 macrophages, from lung adenocarcinoma patients.^206^ Although the glutamine metabolism pattern of macrophages in cancer has still not been fully characterized, inhibiting glutamine metabolism of macrophages has shown the ability to convert M2 macrophages into M1 macrophages.^207^ The serine synthesis of macrophages has been reported to be upregulated in the TME through a protein kinase RNA-like ER kinase-signaling cascade, enhancing mitochondrial function and M2 macrophage activation.^208^

In the TME of triple-negative breast cancer, effector T cells competitively consume glutamine with cancer cells, and the high glutamine metabolism level in tumors is associated with decreased cytotoxicity of T cells.^209^ In lung cancer, blocking glutamine metabolism can improve anticancer immunity by increasing the infiltration of CD8^+^ T cells and CD4^+^ Th1 cells.^210^ Serine and glycine are elevated in mice with T-cell acute lymphoblastic leukemia, which is related to the upregulation of phosphoserine phosphatase.^211^

Studies on amino acid metabolism of other immune cells in cancer are still limited. In colorectal cancer, a subset of immunoregulatory B cells with leucine nutrient preference is correlated with poor survival.^130^ Otherwise, there are more studies on amino acid related signaling and treatments targeting amino acid metabolism to treat cancer, which is going to be discussed in the following sections.

#### Effects of metabolites on immune cells in cancer

Although it is important for understanding immunometabolism in cancer, studies on the changed metabolic pathways of immune cells in cancer are still limited. Some studies have shown that metabolites from the TME can influence the functions of immune cells, which can also enhance our understanding of immunometabolism in cancer. The metabolic state of the TME is disordered. The levels of many metabolites increase in the TME, contributing to the immunosuppression.

Lactate is an important immune inhibitory metabolite generated by tumor cells.^212–214^ Polarization of macrophages in the TME can be induced by lactate, the transport of which depends on mitochondrial pyruvate carrier.^215^ Researchers believe that lactate, rather than its downstream metabolites results in the M2 polarization.^215^ In pituitary adenoma, lactate induces M2 polarization through the mTORC2 and ERK pathways, after which the M2 macrophages secrete CCL17 to promote invasion.^216^ In colorectal cancer, methylation of MCT1, a monocarboxylate transporter protein, can promote M2 polarization and lactate shuttling.^217^ Odorant receptors also participate in M2 polarization in cancer.^218^ After polarization, the functions of macrophages can be influenced by lactate. The expression of the macrophage-specific vacuolar ATPase subunit ATP6V0d2 can be downregulated by lactate, enhancing the production of VEGF mediated by HIF-2α and promoting cancer growth.^219^ In glioma, GPR65 is highly expressed on macrophages, which can sense the stimulation of lactate in the TME and then secrete HMGB1 to promote glioma progression.^220^ Lactate generated from tumor cells also regulates the activation and function of T cells, weakening the immune surveillance.^221–224^ Lactate can enhance the function of Treg cells. Lactate-enhanced MOESIN lactylation and TGF-β signaling in Treg cells can promote tumorigenesis.^225^ Lactate can also regulate Foxp3-dependent RNA splicing in Treg cells in the TME via CTLA-4.^226^ Moreover, the cytotoxicity of CD8^+^ T cells can be inhibited by tumor-derived lactate, which is considered as a potential therapeutic target.^227,228^

Amino acids are other important metabolites in the TME.^229^ Glutamine is the most studied amino acid in immunometabolism. Glutamine metabolism is upregulated in trastuzumab-resistant gastric cancer, and tumor-derived glutaminase microvesicles can induce M2 polarization of macrophages.^61^ In clear cell renal cell carcinoma, tumor cells consume glutamine, promoting macrophages to secrete IL-23 through activating HIF-1α and ultimately suppressing tumor-cell killing.^230^ The competition for glutamine uptake with tumor cells inhibits dendritic cells from priming T cells, and the transporter solute carrier (SLC) family 38 member 2 (SLC38A2) is important for this competition.^231^ In patients with natural-killer T-cell lymphoma, tumor cells increase the glutamine uptake through the ectopic expression of SLC1A1, resulting in the reduced activity of CD3^+^ and CD8^+^ T cells.^232^ In HCC, decreased glutamine uptake can impair the function of CD8^+^ T cells by inducing mitochondrial damage and apoptosis.^233^ Other amino acids also regulate the function of immune cells in cancer. In bladder cancer, increased serine synthesis in cancer cells can induce the M2 macrophage polarization by activating the PI3K/Akt pathway in macrophages.^69^ In gastric cancer, the serine protease PRSS23 can promote macrophage infiltration and result in a poor prognosis through FGF2.^234^ Enriched serine in the TME can also promote the accumulation of Treg cells by regulating sphinganine-mediated c-Fos.^235^

### Immunometabolism in autoimmune diseases

**Fig. 4:**
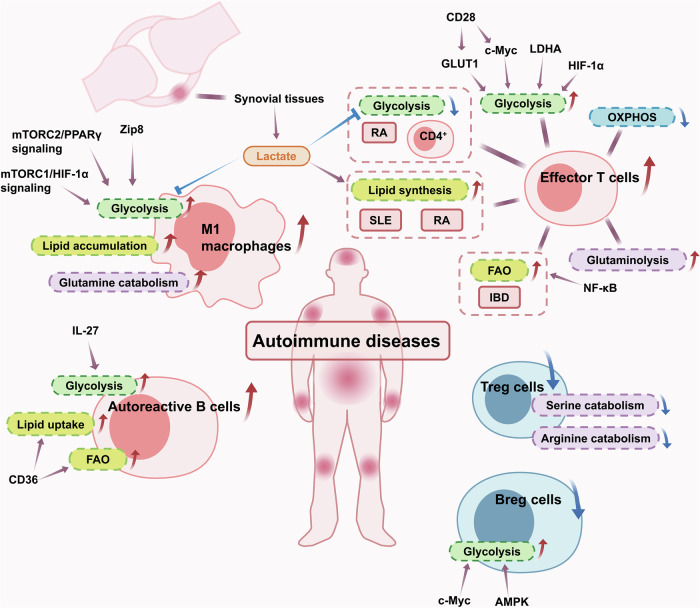
Metabolic regulation of immune cells in autoimmune diseases. Systemic lupus erythematosus (SLE), inflammatory bowel diseases (IBDs) and rheumatoid arthritis (RA) are typical representatives of autoimmune diseases. Generally, affected sites of autoimmune diseases are dominated by immune cells with proinflammatory phenotypes. The metabolism of immune cells with proinflammatory phenotypes are active in the context of autoimmune diseases. Mechanistic target of rapamycin complex 1 (mTORC1)/hypoxia-inducible factor 1α (HIF-1α) signaling, mechanistic target of rapamycin complex 2 (mTORC2)/peroxisome proliferator-activated receptor γ (PPARγ) signaling, Zip8, glucose transporter type 1 (GLUT1), cellular myelocytomatosis oncogene (c-Myc), lactate dehydrogenase A (LDHA), and interleukin-27 (IL-27) have been proved to participate in the regulation of glycometabolism. Nuclear factor-kappaB (NF-κB) and CD36 can regulate fatty acid oxidation (FAO). In RA, lactate from synovial tissues is involved in regulating the metabolism of immune cells

Inflammation is involved in multiple autoimmune diseases. Figure 4 summarizes the metabolic regulation of immune cells in autoimmune disorders.

#### Immunometabolism in systemic lupus erythematosus

Systemic lupus erythematosus (SLE) is a prototypical autoimmune disease characterized by the dysregulation of many immune cells including autoreactive B cells, macrophages, CD4^+^ T cells, dendritic cells, and neutrophils.^236^ The altered metabolic patterns of immune cells in SLE have been reported and are considered as potential therapeutic targets.^237,238^

In the context of SLE, glycometabolism is upregulated in the proinflammatory immune cells.^85^ Glycolysis of human and mouse macrophages can be induced by IgG immune complex and depends on mTOR and HIF-1α, resulting in the upregulation of IL-1β.^239^ Activated lymphocyte-derived DNA, which can induce M2b polarization of macrophages and has been reported to be an important factor in the development of SLE, can also enhance glycolysis and glycogenesis and downregulate the PPP.^240^ Notably, M2b macrophages are considered to be anti-inflammatory, but additional studies are needed to validate these conflicting results.^241^ The cellular metabolism of CD4^+^ T cells, including glycolysis, in SLE patients and mouse is overactivated, and CD4^+^ T cells are considered key immune cells for treating SLE through the regulation of cellular metabolism.^242–244^ Inhibitors of glycolysis can block glucose uptake of CD4^+^ T cells to attenuate autoimmune activation.^242,245^ In Treg cells, the impaired immunosuppressive functions in SLE can be enhanced by exogenous phosphofructokinase P through phosphorylating the glycolysis rate-limiting enzyme 6-phosphofructokinase and upregulating aerobic glycolysis.^246^ The differentiation of follicular helper T cells in SLE mice has been proven to be related to glycolysis and the PKM2 may be a key factor in SLE pathogenesis.^247,248^ B cells have also been considered to play a role in the glucose metabolic dysfunction in SLE mice.^249^ Breg cells, which secrete anti-inflammatory IL-10, can be changed the phenotype into aggressive inflammatory phenotype by upregulated cellular Myc (c-Myc) and enhanced glycolysis through the MAPK signaling in SLE.^250^

Lipid metabolism in immune cells from SLE patients or mice is changed, and lipid metabolism is considered as a potential therapeutic target, but research on lipid metabolism is insufficient.^251^ FcgRIIB dysfunction is common in SLE patients. In vitro, FcgRIIB^-/-^ macrophages exhibit greater lipid accumulation, indicating that lipid metabolism dysregulation in macrophages may be related to the pathogenic mechanisms of SLE.^252^ Two decades ago, researchers have reported that the composition of lipid rafts on T cells from SLE patients is changed, causing aberrant autoimmune responses.^253,254^ However, besides lipid signaling, studies on lipid metabolism in T cells are still limited. Generally, the lipid synthesis in T cells from SLE patients is enhanced, favoring the function of proinflammatory Th17 cells.^85^ As the essential cells for the development of SLE, autoreactive B cells might have enhanced lipid uptake mediated by CD36 and upregulated FAO, and during this process, acetylcholine from spleen fibroblastic reticular cells is important.^255^

Amino acid metabolism of immune cells in SLE is also important, but related studies in the context of SLE are limited.^256^ Limited evidences have shown that glutaminolysis of T cells in SLE is enhanced, helping Th17 cells to differentiate.^85^ Glutaminase 1 inhibition can inhibit CD4^+^ T cells from SLE patients from differentiating into Th17 cells, and simultaneously inhibit the expression of HIF-1α and downregulate glycolysis.^257,258^ The absence of glutamine can inhibit OXPHOS and plasmablast differentiation in peripheral B cells from SLE patients.^124^ Although summarizing the metabolic patterns of amino acids in immune cells from SLE patients is difficult, the importance of related signaling molecules in SLE is undoubted. The expression of serine/threonine protein phosphatase 2 A (PP2A) is increased its expression in SLE patients, resulting in decreased IL-2 and increased IL-17 in T cells, which induces the development of glomerulonephritis.^259^ The regulatory subunit PPP2R2A is upregulated in T cells from SLE patients and can enhance Th1 and Th17 differentiation by activating the GEF-H1/RhoA/ROCK pathway.^260^ However, the production of PPP2R2B is reduced in SLE patients, helping T cells become resistant to apoptosis.^261^ Increased activity of PP2A has also been observed in B cells from SLE mice and patients, and is related to the expression of purine nucleoside phosphorylases.^262^ Another important factor, serine/arginine-rich splicing factor 1 (SRSF1), is downregulated in T cells from SLE patients, and the ubiquitination of SRSF1 is increased, which is also related to decreased IL-2.^263,264^ In SRSF1-deficient mice, the activity of mechanistic targets of mTORC1 is upregulated, indicating a potential therapeutic target for reducing the activity of T cells in SLE.^265^ Decreased SRSF1 levels are also correlated with lymphopenia in SLE patients, which is associated with decreased expression of the anti-apoptotic protein Bcl-xL.^266^

#### Immunometabolism in inflammatory bowel disease

Inflammatory bowel diseases (IBDs), including ulcerative colitis and Crohn’s disease, has spread globally. The spread of Western diet patterns contributes to the prevalence of IBDs, leading researchers to consider the roles of metabolism and immunometabolism in IBDs.^267^

Like in SLE patients, glycometabolism in immune cells from IBD patients is dominated by the cells with a proinflammatory phenotype. M1 macrophage polarization is upregulated in IBD. mTORC1/HIF-1α and mTORC2/PPAR-γ signaling have been shown to be associated with aerobic glycolysis of macrophages from mice with ulcerative colitis.^267,268^ T cells are excessively activated in IBDs.^269^ Impaired mitochondrial respiration has been shown in colitogenic T cells.^270^ In ulcerative colitis, HIF-1α also mediates glycolysis in Th17 cells, and glycolysis in Treg cells is associated with the aryl hydrocarbon receptor.^271,272^ In patients with acute severe ulcerative colitis, HIF-1α expression and glycolysis are also increased in neutrophils.^273^

Lipid metabolism contributes to in the development of therapies for IBD.^274^ However, in the context of IBD, changes in lipid metabolism of immune cells have not been fully described, with only a few studies reporting possible factors or pathways regulating lipid metabolism in immune cells. Fatty acid binding protein 5 can regulate lipid metabolism, which is upregulated in the mucosa of IBD patients.^275^ Inhibiting fatty acid binding protein 5 can promote the M2 polarization of macrophages and plays a protective role against colitis.^275^ Increased FAO has been found in tissue-resident memory CD4^+^ T cells from patients with Crohn’s disease, which induces a proinflammatory phenotype, and the activated NF-κB signaling participates in the upregulated lipid metabolism.^276^

The roles of signaling molecules related to amino acids in the development of IBDs have been reported. Members of the receptor-interacting serine/threonine kinase (RIPK) family are typical representatives. Loss-of-function mutations in RIPK1 are related to immunodeficiency and IBDs, which are also related to reduced NF-κB activity and abnormal differentiation of T and B cells.^277^ The expression of RIPK2 is upregulated in the colonic mucosa of IBD patients, and is important for NOD2 signaling.^278,279^ In intestinal biopsy specimens from IBD patients, inhibiting RIPK2 can decrease proinflammatory cytokine release.^280^ The inhibition of RIPK3 can downregulate proinflammatory cytokine expression in mononuclear cells from ulcerative colitis patients.^281^ Other molecules have also been reported. The upregulation of leucine-rich repeats containing X1 can protect mice from IBDs, and in vitro, leucine-rich repeats containing X1 can decrease the differentiation of CD4^+^ T cells into Th1 and Th17 cells and increase OXPHOS.^282^ The deficiency of leucine-rich repeat kinase 2 can enhance susceptibility to colitis in mice.^283^

The gastrointestinal tract is both an immune organ and a digestive organ. Different from other diseases, in the context of IBDs, researchers have focused on changes in metabolism patterns in a specific organ, the gastrointestinal tract. Glycolysis in intestinal samples from IBD patients has been observed to be significantly upregulated compared with that in intestinal samples from healthy people, which is associated with the upregulated expression of 6-phosphofructo-2-kinase/fructose-2, 6-bisphosphatase 3.^284^ Inhibiting 6-phosphofructo-2-kinase/fructose-2, 6-bisphosphatase 3 can reduce the infiltration of immune cells in the intestine.^284^ In intestinal epithelial cells from ulcerative colitis patients, glucose metabolism is increased, but how impaired glucose metabolism homeostasis in intestinal epithelial cells is associated with immune cells is still unclear.^285^ With the increasing recognition that diet is a pivotal factor for IBD development and progression, dietary lipids have been explored for the treatment of IBDs. Generally, short-chain fatty acids are protective factors with anti-inflammatory properties, but some long-chain fatty acids, including saturated fatty acids and trans fatty acids, are proinflammatory.^286^ How dietary lipids are associated with lipid metabolism and immune responses is a valuable topic for IBDs. Commonly increased serum lipids in IBD patients also indicate the considerable roles for lipid metabolism in managing IBD patients, but how the altered lipid metabolism causes an intestinal inflammatory state and IBD progression is unclear.^287^

#### Immunometabolism in rheumatoid arthritis

Rheumatoid arthritis (RA) is also a typical autoimmune disease characterized by immune cell dysfunction. The metabolic patterns of immune cells in RA have several distinct characteristics. Generally, at the site of RA involvement, immune cells are proinflammatory and under metabolic stress, building a glucose-deficient microenvironment that is somewhat similar to the TME.^288–290^

In rheumatoid synovial tissue and peripheral blood from RA patients, some immune cells with high metabolic demands undergo a metabolic shift from OXPHOS to glycolysis.^291^ The lactate level increases in synovial samples from RA patients, with increased glucose uptake and upregulated GLUT1.^292^ In macrophages from RA patients, the expression of Zip8, a zinc-specific importer, is upregulated, which is related to the activation of glycolysis induced by mTORC1, resulting in IL-1β upregulation.^293^ Inhibiting glycolysis in macrophages from RA rats and patients can promote their transition from the M1 phenotype into the M2 phenotype.^294,295^ Changes in the transcriptional regulation of glycolysis in peripheral T cells from RA patients have been shown.^296^ Increased LDHA activity and aerobic glycolysis have been reported in peripheral CD8^+^ T cells from RA patients.^297^ The glycolysis of T cells in RA is associated with ICOSL, which is regulated by B cells and participates in the polarization of Th cells.^298^ Then activated Th cells can upregulate aerobic glycolysis and drive the inflammatory phenotype transition of synovial fibroblasts, resulting in the progression of RA.^299^ Dendritic cells in synovial tissue are activated in RA, with enhanced glycolysis and anabolism, and their ability to activate T cells is enhanced.^300^ In contrast to CD8^+^ T cells, CD4^+^ T cells from RA patients exhibit decreased glycolysis and upregulated PPP.^289^ Glycolysis of peripheral B cells from RA patients is also increased, and IL-27 is a possible upstream activating factor.^301^ Glucose-6-phosphate isomerase, which can catalyze the interconversion between D-glucose-6-phosphate and D-fructose-6-phosphate, can induce chronic arthritis via B cells in mice to establish a type of RA model.^302^

Lipid metabolism in immune cells from RA patients is associated with the severity of RA. Data from the Gene Expression Omnibus database have shown that the expression profiles of fatty acid metabolism-related genes in peripheral CD8^+^ T cells are different between RA patients and healthy people, and the expression of fatty acid metabolism-related genes including FABP4 and GPR84 are upregulated the expression in RA patients who respond well to methotrexate.^303^ In mice with collagen-induced arthritis, the level of α2-glycoprotein 1, a protein that stimulates lipolysis, is increased, increasing the Th17 population of splenocytes and exacerbating RA.^304^

Lactate has been considered as a junction of metabolism, inflammation, and autoimmunity.^305^ Dysregulated lactate metabolism and lactate accumulation in the synovial tissue are important characteristics of RA. Local hypoxia at the involved sites is a main cause of lactate accumulation.^306^ The functions of both synovial fibroblasts and immune cells can be affected by lactate in RA. Genes regulating lactate intake and secretion can be upregulated upon inflammation, and the members SLC16A1 and SLC16A3 are expressed in synovial fibroblasts and macrophages.^307^ Lactate can upregulate glycolysis in fibroblasts and downregulate glycolysis of macrophages in the synovial tissue of individuals with RA.^307^ The lactate transporter SLC5A12 in CD4^+^ T cells can be upregulated by lactate, increasing IL-17 production and fatty acid synthesis and decreasing glycolysis in CD4^+^ T cells, thus promoting the development of RA.^308,309^ The Warburg effect is upregulated in CD8^+^ T cells from RA patients, and LDHA is overexpressed, participating in the induction of the proinflammatory phenotype of B cells.^297^

#### Immunometabolism in multiple sclerosis

Multiple sclerosis (MS) is characterized by the chronic inflammation of the central nervous system, which causes neurological disability in young adults.^310,311^ Multiple immune cells, especially T cells, participate in the development and progression of MS.^312^

Changes in glucose metabolism in immune cells contribute to inflammatory responses in MS. Glycolysis in CD4^+^ T cells can be upregulated by the CD28-mediated c-Myc and GLUT1 upregulation in relapsing-remitting MS patients, which is important for the production of inflammatory cytokines by Th17 cells.^313^ However, some researchers believe that glycolysis and OXPHOS of T cells in relapsing-remitting MS patients are impaired, as determined by measuring the extracellular acidification rate and oxygen consumption rate, but they also reported the important role of GLUT1.^314^

Lipid metabolism of immune cells has long been considered as a promising therapeutic target for MS, but related studies are still limited.^315^ RNA-sequencing analysis has revealed the dysregulation of lipid metabolism genes in CD4^+^ T cells from relapsing-remitting MS patients, with liver X receptors mediating lipid metabolism pathways.^316^ As the most important cells for the clearance of myelin, the lipid metabolism of macrophages might change in MS, which also deserves attention.^317^

Amino acid homeostasis is also impaired in MS. An animal model of MS has shown that glutaminase is upregulated in macrophages near dystrophic axons.^318^ Transglutaminase has been found to be expressed in macrophages but not in T cells or B cells, indicating its possible role in macrophage infiltration into the central nervous system.^319,320^ The peripheral blood mononuclear cells of MS patients have exhibited decreased activity of enzymes involved in tryptophan and arginine catabolism, resulting in a decrease in the number of Treg cells.^321^

### Immunometabolism in metabolic diseases

**Fig. 5:**
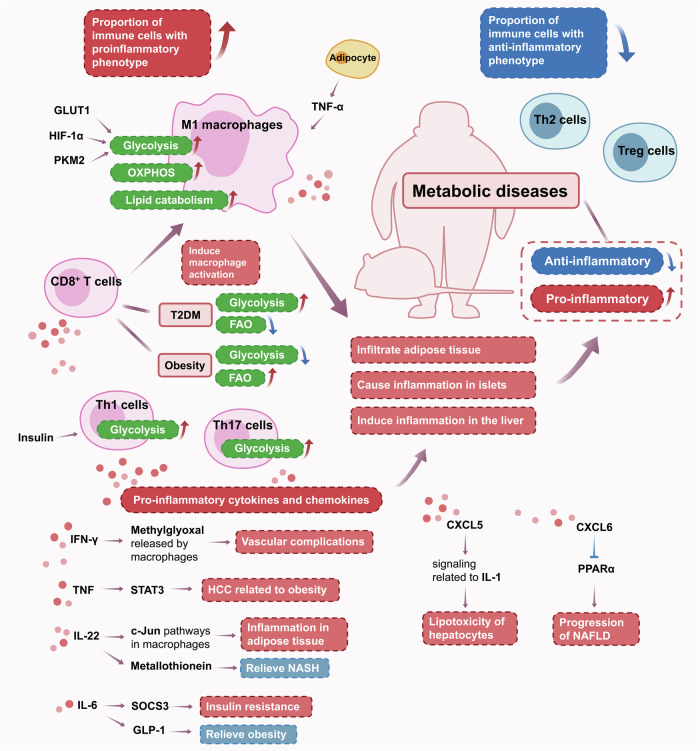
Metabolic regulation of immune cells in metabolic diseases. Chronic low-grade inflammation has been considered as an important trait of metabolic diseases. Obesity, type 2 diabetes mellitus (T2DM) and non-alcoholic fatty liver disease (NAFLD) are typical representatives of metabolic diseases. Tumor necrosis factor (TNF)-α from adipocytes can induce M1 macrophage polarization, and insulin supports interleukin (IL)-1-producing T helper (Th1) cells cell differentiation. Generally, in immune cells with proinflammatory phenotype, the metabolic pathways are upregulated. Glucose transporter type 1 (GLUT1), Hypoxia-inducible factor-1α (HIF-1α) and pyruvate kinase muscle isozyme M2 (PKM2) are involved in regulating the metabolism of immune cells. After being activated, M1 macrophages can infiltrate adipose tissue, cause inflammation in islets and induce inflammation in the liver. Pro-inflammatory cytokines and chemokines produced by immune cells participate in disease progression

Chronic low-grade inflammation in patients with metabolic diseases has been considered as an important trait. In Fig. 5, we have summarized the changes in phenotypes and metabolism of immune cells, and cytokines affecting disease progression in metabolic diseases.

#### Immunometabolism in obesity

Obesity, always accompanied by the accumulation of body fat, increases the risk of diabetes, non-alcoholic fatty liver disease (NAFLD), and many other diseases.^322^ Chronic low-grade inflammation in the adipose tissue is an important characteristic of obesity, motivating researchers to explore the causal relationship between immune responses and obesity.^323^ The functions of immune cells can be regulated by cellular metabolic reprogramming.^324^

Many immune cells exhibit a proinflammatory phenotype in individuals with obesity. Macrophages are abundant immune cells in adipose tissue.^325^ In obese individuals, macrophages in adipose tissue switch from the M2 phenotype to the M1 phenotype, and M1 macrophages infiltrate adipose tissue.^326–328^ As the proinflammatory phenotype, M1 macrophages release cytokines and chemokines to initiate inflammatory responses in obese individuals.^329^ Many studies have reported changes in the activation and function of T cells in obesity and obesity-related pathological conditions. The anti-inflammatory environment predominates in healthy adipose tissues.^330^ In obesity, the levels of anti-inflammatory T cell subsets are decreased, including Th2 and Treg cells, while the numbers and proportions of proinflammatory effector T cells, including Th1 and Th17 cells, in adipose tissue and in the circulation are increased.^331^

Accompanied by changes in the proinflammatory phenotype, immune cells also change their intracellular metabolic patterns. First, we discuss the glycometabolism of immune cells in obesity. Increased glycolysis and OXPHOS of adipose tissue macrophages from obese mice are important metabolic characteristics of macrophages under conditions of obesity.^324^ The increased glucose uptake and metabolism of macrophages from obese individuals is related to GLUT1 and HIF-1α, which promote the production of IL-1β, leading to oxidative stress and the proinflammatory response.^324,332,333^ In obese individuals, glycolysis of T cells is upregulated and CD4^+^ T cells are overactivated.^334^ Insulin increases the glucose uptake into the cells to upregulate glycolysis of T cells, supporting Th1 cell differentiation.^330^ Obesity increases B cell recruitment to adipose tissue, indicating the importance of B cells in obesity, but knowledge of glycometabolic patterns of B cells in obesity is still lacking.^335^

In obesity, lipid metabolism of immune cells is modified. Obesity promotes macrophages to induce intracellular lipid catabolism, which depends on lysosomes.^336^ The FAO of CD8^+^ T cells is upregulated in obese mice with breast cancer through the activation of STAT3, which is also related to decreased glycolysis of CD8^+^ T cells.^337^ The FAS of Th17 cells from obese mice is regulated by acetyl-CoA carboxylase 1, then regulating the function of RORγt.^338^ The FAO of dendritic cells from obese mice is upregulated, promoting the intracellular ROS accumulation and impairing antigen presentation.^339^ In addition to the changes in lipid metabolism of immune cells, changes in lipid metabolism of the obese individual are also important for regulating immune cell activities. In obese individuals, adipocytes release large amounts of fatty acids which can increase tumor necrosis factor (TNF)-α, which can induce M1 macrophage polarization.^340^

Changes in the levels of cytokines and chemokines in obesity have received some attention. Because metabolic diseases are known as disorders of metabolism, immunometabolism in metabolic diseases sometimes does not mention metabolic pathways in specific cells. Considering their important functions in regulating immune cell function, cytokines are also involved in immunometabolism in obesity. For chronic inflammation in obesity, IL-6 is one of the most discussed mediators, and whether IL-6 is protective or nonprotective to obese individuals is still controversial.^341^ The level of IL-6 increases in obese patients.^341^ IL-6 can increase islet GLP-1 production to attenuate obesity and exogenous IL-6 reduces diet-induced obesity in mice, but IL-6 also worsens insulin resistance in the liver and adipose tissue.^342,343^ Other cytokines, including IFN-γ and TNFs, are upregulated in obese individuals, promoting chronic inflammation and worsening the prognosis.^344–346^ The expression of CC chemokines and CXC chemokines in visceral adipose tissue is upregulated in obese patients and is associated with increased inflammation.^347–350^

#### Immunometabolism in T2DM

Chronic and low-grade inflammatory disease with long-term immune system imbalance is also a characteristic of T2DM.^351^

The proinflammatory phenotype is the dominant phenotype of immune cells in T2DM. In T2DM, M1 macrophages are the dominant immune cells that cause inflammation in islets.^352–354^ The expression levels of PPARγ in macrophages from T2DM patients are decreased, and macrophage-specific PPARγ controls M2 macrophage activation.^355,356^ The proportion of CD4^+^ T cells increases and the proportion of CD8^+^ T cells decreases after glucose loading in people with or without diabetes, indicating a more active state for CD4^+^ T cells in T2DM.^357^ Th1 and Th17 cells are proinflammatory subsets of CD4^+^ T cells, while Th2 and Treg cells are anti-inflammatory subsets of CD4^+^ T cells.^358^ The numbers of Th1 and Th17 cells increase in T2DM patients.^359,360^ IL-17 released by Th17 cells plays a key role in inflammation, insulin resistance, and T2DM, with harmful effects on T2DM patients.^361^ The percentage of circulating Th2 cells is inversely correlated with insulin resistance.^362^ The percentage of peripheral Treg cells decreases in T2DM patients.^363^ CD8^+^ T cells can also secrete chemokines to induce macrophage activation, but the proinflammatory mechanisms involved in T2DM or insulin resistance are still unclear.^351^

Related studies on obesity can focus on immune cells in adipose tissues, but studies on T2DM always focus on the general symptoms, making it difficult to focus on a specific organ or tissue. In the context of T2DM, studies on the metabolic pattern of immune cells are very limited. Macrophages extracted from the peritoneum of T2DM mice exhibit decreased glucose uptake and glycolysis with downregulated GLUT1.^364^ Moreover, peripheral T cells from T2DM patients prefer glycolysis instead of FAO to obtain energy.^365^

As T2DM is a metabolic disease, changes in cytokines levels might be a simpler topic for researchers to associate metabolism with immunity in T2DM. IL-6 is also one of the most discussed mediators of T2DM. The role of IL-6 in diabetes has been discussed in a previous review.^366,367^ Recent studies have shown that IL-6 can suppress the expression of suppressor of cytokine signaling-3 to impair the phosphorylation of insulin receptors, leading to insulin resistance.^368^ The level of plasma IL-6 is positively correlated with the risk of the cardiovascular and kidney outcomes in T2DM patients.^369,370^ A small number of ILs have been proven to be protective against T2DM. As a prominent anti-inflammatory cytokine, IL-10 reduces neurogenic inflammation in a T2DM mouse model.^371^ More studies have reported the nonprotective effects of ILs in T2DM. IL-1, a cytokine family with proinflammatory functions, its signals can promote the progression of insulin resistance and diabetes, which has been summarized in a previous review.^372,373^ IL-2 and IL-18 play proinflammatory roles and have been shown increased levels in T2DM.^374,375^ The levels of serum IL-19, IL-38, and IL-39 are positively correlated with the risk of diabetic complications.^376–378^ IFN-γ plays non-protective roles in T2DM patients, but the underlying mechanisms are still unclear. A decrease in the level of IFN-γ decreases in the serum of T2DM patients is associated with increased oral candidiasis incidence, increased risk of tuberculosis and infection of diabetic ulcers.^379–382^ The serum TNF-α concentration is increased in T2DM patients.^383^ In T2DM patients, urinary TNF-α levels, but not serum TNF-α levels, are associated with the progression of nephropathy.^384,385^ Upregulated chemokine levels are nonprotective to T2DM patients.^386,387^ The increased chemokine levels are possibly caused by LPS stimulation in T2DM patients, which can predict hepatic steatosis.^388,389^

#### Immunometabolism in NAFLD

With the increasing prevalence of obesity and diabetes, the prevalence of NAFLD is increasing.^390^ During the variable course of NAFLD, ignorable hepatic lipid accumulation and liver inflammation are developed.

Immune cells play important roles in the pathological processes of NAFLD. In NAFLD, macrophages are activated and changed to the proinflammatory phenotype in NAFLD. Macrophages play important roles in the pathological processes of NAFLD. In a healthy liver, Kupffer cells secrete the anti-inflammatory cytokine IL-10 to provide an anti-inflammatory microenvironment.^391^ In NAFLD, macrophages can be activated by endotoxins, fatty acids, cholesterol and any other stimuli.^392^ Then, macrophages, including Kupffer cells, participate in inducing inflammation in NAFLD by releasing cytokines such as IL-1β and TNF-α.^393–395^ The expression of TLR4 is also upregulated in NAFLD patients.^396^ The TLR4-MyD88-mediated NF-κB and MAPK pathways can induce excessive immune responses in NAFLD.^397^ The activation of macrophages, which is a key event in fibrogenesis, can promote liver fibrosis and HCC in NAFLD through complex mechanisms that have been fully discussed in some reviews.^396,398^ Generally, macrophages are an integral part of the hepatocyte-macrophage-hepatic stellate cell network in NASH and are involved in signal transduction.^398^ The mechanism by which T cells are activated by metabolic dysregulation in NAFLD has not been fully elucidated. Lipid accumulation in hepatocytes initiates the pathogenesis of NAFLD and subsequently increases the release of reactive oxygen species (ROS) and free lipids.^399^ It can be noted that ROS have been considered as important signaling messengers to regulate the activation of T cells.^400,401^ The liver has been considered as an immunological organ for a long time, with B cells being the most abundant intrahepatic lymphocytes.^402,403^ Although the subsets of B cells in the liver tissue of NAFLD patients are unclear, B2 cells are the main B cell populations in the liver tissue of NASH mice.^404^ With the progression of NAFLD, serum B cell-activating factor levels increase.^405,406^ Nevertheless, whether the accumulation and activation of B cells and B2 subsets are causal or consequential to NASH is still unclear.^407^ It is relatively clear that B cells promote the progression of NAFLD. In the early stages of NAFLD, B2 cells are activated.^408^ During NAFLD, intrahepatic B cells express proinflammatory genes, secrete IL‐6 and TNF-α and subsequently promote a chronic inflammation and fibrogenesis.^404,409^

Studies on the metabolic patterns of immune cells in NAFLD are limited, but can indicate changes in metabolic patterns. In high-fat-diet induced mice, glycolysis and OXPHOS of macrophages are upregulated, with caspase-11 and PKM2 are involved in the upregulation of glucose metabolism.^410–413^ The lipid uptake and accumulation of Kupffer cells are increased in NAFLD, which can be mediated by macrophage scavenger receptor 1, leading to liver inflammation.^393^

Cytokines are also the entry point for immunometabolism in NAFLD. The levels of IL-6, IL-12, IL-32 and IL-38 in the blood are upregulated in NAFLD patients, with positive correlations to severity of NAFLD.^414–417^ In NASH mice, inhibiting IL-11 signaling can reduce inflammation and liver fibrosis, possibly due to the ability of IL-11 to activate myofibroblasts, indicating the nonprotective role of IL-11.^418,419^ Blocking the IL-6 receptor can increase the risk of NAFLD, indicating the protective role of IL-6 in NAFLD.^420^ IL-17 can promote microbiota-related intestinal barrier restoration to alleviate NASH in high-fat-diet induced NASH mice, but increased IL-17A correlates positively with steatosis in humans with NASH.^421,422^ IL-22 alleviates NASH by blocking hepatic oxidative stress through the induction of metallothionein.^423^ The role of IFN-γ in NAFLD are controversial. Type I IFN responses can drive T cells in the liver to promote metabolic syndrome.^424^ Treating NAFLD mice with IFN-α showing aggravated liver fibrosis.^425^ However, adipose-specific deletion of IFN-α and IFN-β receptors promotes metabolic dysregulation in NAFLD mice, which indicates possible protective roles of IFN in NAFLD.^426^ The level of TNF-α also increases in NAFLD individuals.^427^ TNF-α is considered as a central player in liver inflammation with multiple functions, and overall, it is a harmful molecule to the liver.^428^ Inhibiting the receptor of TNF-α can reduce liver steatosis, hepatocellular injury and fibrosis in NAFLD mice.^429^ The levels of chemokines are upregulated in NAFLD patients, with unclear mechanisms but clear results indicate the harmful roles of chemokines in NAFLD.^430,431^ Chemokines promote the development of steatosis, NASH, and fibrosis, as discussed in previous reviews.^432–435^ Recent studies have shown that CCL3 can promote liver macrophage infiltration and M1 polarization during the progression of NAFLD.^436^ The expression of CCL20 increases with the NAFLD stage, and can be regulated by microRNA-590-5p.^437^ Inhibiting CCL11 in NAFLD mice can reduce immune cell infiltration and liver fibrosis, with IFN regulatory factor 1 serving as a mediator.^438^ Inhibiting CCR9 also reduces fibrosis progression in NASH mice.^430^ The highly upregulated expression of CXCL1 in the liver can drive steatosis to NASH through neutrophil-derived ROS.^423^ CXCL5 can promote lipotoxicity in hepatocytes in mice with NASH, potentially through the regulation of signaling related to IL-1 in Kupffer cells.^439^ Inhibiting CXCL6 can upregulate PPARα in cell models of NAFLD.^440^ In NAFLD mice, CXCL10 can mediate the polarization of M1 macrophages and macrophage infiltration in the liver.^441^ In summary, although the mechanisms are still unclear, especially in humans, the results of recent studies support the supposition that chemokines play harmful roles in NAFLD and can be potential therapeutic targets.

### Immunometabolism in infection

During infection, immune cell activity substantially changes. Metabolic reprogramming of immune cells also occurs upon infection, determining the pathogen growth or containment.^442^

In most cases, glycometabolism of immune cells is positively correlated with anti-infectious immunity.^443^ Upon Brucella infection, glycolysis of macrophages is upregulated by MyD88 to control Brucella infection.^444^ Reduced glycolytic activity is an important characteristic of exhausted CD8^+^ T cells in HIV-infected individuals, with dysregulated mTOR signaling.^445^ Glycolysis of CD4^+^ T cells is increased upon HIV-1 infection, with upregulated expression of GLUT and hexokinase 1.^446^ Blocking the glycolysis can increase HIV-1 reverse transcription in CD4^+^ T cells.^447^ On NK cells from HIV-infected individuals, the expression of CD160, which can upregulate glucose uptake through the PI3K/Akt pathway to enhance NK cell function, was reduced.^448^ Upon *H. pylori* infection, glycolysis of macrophages can be upregulated by HIF-1α, which then stabilizes Nos2 messenger RNA to produce nitric oxide and control infection.^449^ However, the upregulated glycometabolism of immune cells can also promote the progression of infection. Upon SARS-CoV-2 infection, glycolysis of monocytes and macrophages is upregulated by ROS and HIF-1α, directly inhibiting the T-cell response.^450^

Lipid metabolism of immune cells is complex and important to immune responses, existing evidence has revealed the roles of FAS. Upon *Mycobacterium tuberculosis* infection, the FAS of macrophages and dendritic cells is induced to mediate the innate response against mycobacteria.^451^ Upon *Plasmodium chabaudi* infection, inhibiting FAS during T-cell priming rather than the survival phase can reduce the number of memory T cells.^452^ Related studies on FAO are more limited than those on FAS. Lipid peroxidation caused by selenoenzyme glutathione (GSH) peroxidase 4 knockout suppresses the expansion of effector T cells.^453^

In the context of infection, studies have reported that the metabolism of glutamine in immune cells can influence immune functions.^454^ Glutamine is an important carbon and nitrogen source for the metabolic reprogramming of *Mycobacterium tuberculosis* induced M1 macrophage polarization, which is a complementary metabolic program of M1 macrophages in addition to HIF-1α mediated glycolysis upregulation.^455^ In vitro, glutamine deficiency and glutamine pathway inhibition can reduce the cytokine responses of mononuclear cells and T cells stimulated with *Mycobacterium tuberculosis*, including IFN-γ, IL-1β, IL-17 and IL-22.^456^ At the early stage of HIV-1 infection, the TCA cycle and OXPHOS of naive T cells and memory CD4^+^ T cells are majorly fuelled by glutaminolysis, which favors HIV-1 retrotranscription.^447^

### Immunometabolism in diseases of specific systems and organs

Immunometabolism has inspired novel therapies for many diseases, beyond cancer, autoimmune diseases, metabolic diseases, and infection. Here, we summarize some other representative diseases according to the involved and lesion sites.

#### Immunometabolism in cardiovascular diseases

Immune cells carry out functions in the myocardium, that can be regulated by intracellular metabolism and metabolites of cardiac parenchymal cells, motivating researchers to focus on immunometabolism in cardiovascular diseases.^457^

Normal glucose metabolism of immune cells is impaired in cardiovascular diseases. The glycolysis of naive CD4^+^ T cells from mice with atherosclerosis is downregulated, impairing the proliferation and activation of CD4^+^ T cells.^458^ Upon atherosclerosis and myocardial ischemia-reperfusion, glycolysis of macrophages is upregulated, promoting M1 polarization and increasing the levels of inflammatory cytokines.^459,460^ At the early stage of myocardial infarction, glycolysis of macrophages is upregulated and macrophages are mainly in the M1 phenotype, while at the late stage, glycolysis decreases to basal levels, and macrophages are mainly in the M2 phenotype.^461^ Additionally, at the late stage of myocardial infarction, the activity of the PPP in macrophages is upregulated.^461^

The lipid metabolism of macrophages is critical in atherogenesis. Macrophages engulf lipid droplets to form foam cells, populating atherosclerotic plaques, which indicates that lipid uptake by macrophages in atherosclerosis is significantly upregulated.^462–464^ However, the activity of lipid metabolism including FAO in macrophages is insufficient, resulting in lipid accumulation and the development of atherosclerosis.^465,466^ Excessive lipid accumulation has also been observed in the dendritic cells of atherosclerotic lesions.^467^ The lipid metabolism patterns of other immune cells in cardiovascular diseases have not been reported.

Although studying amino acid metabolism seems to be more difficult than studying glucose and lipid metabolism, some studies have reported amino acid metabolism or related signals. Leucine is a key activator of mTOR in macrophages and can drive atherosclerosis in mice fed a high-protein diet.^468,469^

#### Immunometabolism in neurological disorders

Microglia are a specialized macrophages in the central nervous system.^470^ Due to the important roles of microglia in neurological disorders, immunometabolism in nervous system diseases has been noted by researchers, with Alzheimer’s disease as a representative disease.^471^

In multiple neurological disorders, immune cells are changed into a proinflammatory phenotype, with metabolic reprogramming occurring. In Alzheimer’s disease, a decrease in M2b macrophages has been considered an indicator of cognitive function.^472^ Microglia are activated into the proinflammatory phenotype, with a switch from OXPHOS to glycolysis.^473^ The glucose uptake of microglia is positively correlated with their activity.^474^ Nevertheless, in neurodegenerative disorders, the glucose uptake of microglia is upregulated, but glucose hypometabolism occurs in the affected region of the brain.^475^

Lipid metabolism of microglia differs between individuals with and without Alzheimer’s disease.^476,477^ Abnormal accumulation of lipid droplets has been observed in microglia from individuals with Alzheimer’s disease, with downregulated expression of BHLHE40/41.^478,479^ A recent study believes that the APOE4 genotype causes the abnormal lipid metabolism of microglia in Alzheimer’s disease, impairing the phagocytic capabilities of microglia and interactions with neurons.^480^

We believe that amino acid metabolism of immune cells may change in neurological disorders based on limited evidence. The release of D-serine can be increased by the inflammatory activation of microglia, which affects synaptogenesis and enhances NMDAR-dependent excitotoxicity, resulting in neurodegeneration.^481^ Two decades ago, a study reported that amyloid β-peptide can induce the release of D-serine in microglia.^482^ When the nervous system is in an inflammatory state, microglia may metabolize glutamate into glutamine instead of requiring the retention of glutamate.^483^

#### Immunometabolism in respiratory disorders

In addition to pulmonary neoplasms and pulmonary infections, immunometabolism also participates in other respiratory disorders. Related studies are limited, and glycometabolism is relatively well-studied.

Upregulated glycolysis and downregulated OXPHOS of M1 macrophages in acute lung injury have been observed, activating NLRP3 inflammasome, and triggering receptor expressed on myeloid cells-1 can mediate this metabolic reprogramming.^484,485^ In acute rejection after lung transplantation, the glucose uptake of immune cells is increased, with CD8^+^ T cells being the largest glucose utilizers.^486^ In pulmonary ischemia-reperfusion injury, glycolysis of activated neutrophils is upregulated, promoting the progression of the disease.^487^

The changes of amino acid metabolism in many respiratory disorders are still unclear, and the results from existing studies prefer to consider amino acids as signal molecules. In lung injuries caused by hyperoxia, the inhibition of glutamine metabolism and decreased GSH are protective to the diseased individuals, but how this regulation affects the activity of immune cells is unclear.^488,489^ Glycine can attenuate acute lung injury by decreasing the upregulation of NLRP3 and increasing the downregulation of NRF2.^490^

## Targets and treatments based on immunometabolism

As previously summarized, immune cells with different phenotypes have different metabolic characteristics, and metabolic pathways can impact the functions of immune cells. The phenotypes and metabolic pathways of immune cells undergo changes when diseases occur and develop, prompting researchers to explore potential therapeutic targets by regulating metabolic pathways (Table 2).

**Table 2:** Targets and treatments based on metabolic regulation of the immune system

Targeted metabolic pathways	Targeted immune cells	Conditions	Treatments	Targets, related pathways or downstream molecules	Research phases	References
↓ glycolysis	Macrophages	Autoimmune diseases, metabolic diseases, atherosclerosis, cardiac ischemia and reperfusion injury, Alzheimer’s disease, acute lung injury	Dioscin, tiliroside, transplantation of mesenchymal stem cells, xanthones from Securidaca inappendiculata Hassk., 2-deoxyglucose, autologous blood transfusion, calenduloside E, salvianolic acid B, sDR5-Fc, PKM2 inhibitor, N-phenethyl-5-phenylpicolinamide, histone methyltransferase SETD2, phloretin, peficitinib, WIN55212-2	• ↓ HIF-1α: mTORC1/HIF-1α pathway • ↓ PKM2: PI3K/Akt/PKM2 pathway • ↓ PFKFB3 • ↓ GLUT1 • ↓ NF-κB • ↓ NAMPT • ↓ ASIC1a • ↓ JAK3/STAT3 pathway • ↑ AMPK • ↑ Kruppel-like transcription factor • ↑ H3K36me3	Preclinical	^268,294,459,460,473,493,495–498,505–507,582,583^
	T cells	Cancer, autoimmune diseases, metabolic diseases	Arginine, JHU083, PFK15, norisoboldine, costunolide, metformin, adiponectin, DC-Gonib32	↓ HIF-1α; ↓ mTOR; ↓ PFKFB3; ↑ AMPK; ↓ JAK/STAT pathway; ↓ NAD/SIRT1/SUV39H1/H3K9me3 pathway, ↓ mitochondrial E3 ubiquitin ligase, ↓ NF-AT signaling,	Arginine, RCT, NCT02017249; metformin, observational study, NCT03960333	^102,271,272,284,334,509,516,518^
	Neutrophils	Acute lung injury	2-deoxyglucose	↓ NLRP3 inflammasome	Preclinical	^487,515^
	Neoplastic lymphocytes	Cancer	HK inhibitor, PP2A, WEE1 inhibitor	↑ glutaminolysis	HK inhibitor, observational study, NCT05610228	^527,528^
↑ glycolysis	Macrophages	Cancer	Vismodegib	↓ Hedgehog signaling, ↓ UDP-GlcNAc biosynthesis pathway	Postmarketing	^520^
	T cells	Cancer	Sirt2 inhibitor, CD28 domain in CAR-T cells, lactate dehydrogenase inhibitor	↓ NR4A	Preclinical	^175,519,521^
	Dendritic cells	Cancer	PFK15 and F16BP	↑ Tc1; ↑ Tc17	Preclinical	^522^
↓ OXPHOS	T cells	Cancer	FABP5 inhibitor	↓ FABP5, ↓ mtDNA, ↓ cGAS-STING-dependent type I IFN signaling, ↓ IL-10	Preclinical	^194^
	Neoplastic lymphocytes	Cancer	IACS-010759	Myc-overexpressing cells	IACS-010759, observational study, NCT02882321	^525^
↑ OXPHOS	T cells	Cancer, autoimmune diseases, metabolic diseases	L-arginine, Foxp3, Sirt2 inhibitor, 4-1BB domain in CAR-T cells, linoleic acid, glutamine antagonist, JHU083, NX-13, metformin	↓ mTOR, ↑ AMPK, ↓ NF-κB, ↓ TNF-α, ↓ glutamine, ↑ calcium signaling, ↑ leucine rich repeat containing X1	Arginine, RCT, NCT02017249; NX-13, RCT, NCT04862741; metformin, observational study, NCT03960333	^79,102,175,210,282,516,519,530,536^
↓ FAO	Macrophages	Cancer	Vismodegib, receptor-interacting protein kinase 3 activator	↓ PPAR, ↓ Hedgehog signaling, ↓ UDP-GlcNAc biosynthesis pathway	Preclinical	^57,520^
	T cells	Autoimmune diseases, metabolic diseases	Carnitine palmitoyltransferase 1 inhibitor	↑ apoptosis	Preclinical	^315^
↑ FAO	Macrophages	Cancer, autoimmune diseases, atherosclerosis, lung inflammation	CD40 activator, Dioscin, angiotensin-converting enzyme, Qingfei oral liquid	↑ PPARα, ↓ ACLY, ↑ CD36, ↑ ABCA1, ↑ABCG1, ↑ mTORC2/PPAR-γ pathway	Preclinical	^465,466,495,538,539^
↓ lipid synthesis	Macrophages	Lung inflammation	Qingfei oral liquid	↑ PPARα, ↓ ACLY	Preclinical	^539^
	T cells	Cancer, autoimmune diseases	SREBP-cleavage-activating protein antagonist, FX11	↓ PIK3, ↓ LDHA	Preclinical	^195,297^
↓ glutamine metabolism	Macrophages	Cancer	Glutamine metabolism inhibitor	↓ CSF3, ↓ kynurenine	Preclinical	^207^
	T cells	Cancer	JPH203, DRP-104, asparaginase	↓ SLC7A5, ↓ SLC1A1, ↓ glutamine-dependent nucleotide synthesis	DRP-104, observational study, NCT06027086	^232,559–562^
	Neoplastic lymphocytes	Cancer	epigallocatechin gallate	↓ glutamine-glutamate-α-ketoglutarate axis	Preclinical	^558^
↑ glutamine metabolism	Macrophages	Cancer	CD40 activator	↑ pro-inflammatory genes	Preclinical	^538^
	T cells	Cancer	V-9302	↑ glutathione	Preclinical	^209^

*PKM2* pyruvate kinase M2, *HIF-1α* hypoxia-inducible factor 1α, *mTORC1* mTOR complex 1, *PI3K* phosphatidyl-inositol 3 kinase, *Akt* protein kinase B, *PFKFB3* 6-phosphofructo-2-kinase/fructose-2,6-bisphosphatase 3, *GLUT1* glucose transporter type 1, *NF-κB* nuclear factor-kappaB, *NAMPT* nicotinamide phosphoribosyltransferase, *ASIC1a* acid-sensing ion channel-1a, *JAK3* Janus kinase 3, *STAT3* transcription 3, *AMPK* AMP-activated protein kinase, *mTOR* mechanistic target of rapamycin, *NAD* nicotinamide adenine dinucleotide, *SIRT1* silent information regulator sirtuin 1, *SUV39H1* suppressor of variegation 3–9 homologue 1, *NF-AT* nuclear factor of activated T cells, *RCT* randomised controlled trial, NLRP3, NACHT, LRR, and PYD domains-containing protein 3. HK hexokinase, *PP2A* protein phosphatase 2A, *WEE1* wee1-like protein kinase, *UDP-GlcNAc* uridine diphospho-N-acetylglucosamine, Sirt2 sirtuin 2, *CAR-T cells* chimeric antigen receptor T cell, *NR4A* nuclear receptor subfamily 4 group A, *PFK15* (E)-1-(pyridin-4-yl)-3-(quinolin-2-yl)prop-2-en-1-one, F16BP fructose 1,6-bisphosphate, *Tc1* type 1 CD8^+^ T cells, *Tc17* interleukin-17-secreting, CD8^+^ T cells, *FABP5* fatty acid-binding protein 5, *mtDNA* mitochondrial genome, *cGAS-STING* cyclic GMP-AMP synthase-stimulator of interferon genes, *IFN* interferon, *IL-10* interleukin-10, *Myc* myelocytomatosis oncogene, *Foxp3* forkhead/winged helix transcriptional factor P3, *TNF-α* tumor necrosis factor-α, *PPAR* peroxisome proliferator-activated receptor, *ACLY* adenosine triphosphate (ATP) citrate lyase, *ABCA1* ATP-binding cassette transporter A1, *ABCG1* ATP-binding cassette subfamily G member 1, *mTORC2* mTOR complex 2, *SREBP* sterol responsive element binding protein, *PIK3* phosphoinositol-3-kinase, *LDHA* lactate dehydrogenase A, *CSF3* colony-stimulating factor 3, *SLC7A5* solute carrier family 7 member 5, *SLC1A1* solute carrier family 1 member 1

### Targeting glycolysis of immune cells to treat diseases

Glycolysis is one of the most studied metabolic pathways in immune cells, with the high metabolic flux representing the activated status of immune cells that is typically seen in proinflammatory immune cells.

To reduce inflammation, downregulating glycolysis of immune cells has been studied, which can be suitable for treating autoimmune diseases, metabolic diseases and other inflammatory diseases. HIF-1α is the factor received most attention, and its activation can upregulate glycolysis in immune cells.^491^ HIF-1α is one of the two subunits of HIF-1, activated in oxygen-free environments, and plays important roles in oxidative stress, cancer progression, and many other diseases.^492–494^ In vitro and in vivo experiments, researchers have tried to suppress HIF-1α to reduce the glycolysis of immune cells, including macrophages and T cells.^268,271,493,495–498^ Inhibitors of HIF-1α are numerous, most of which have been used for macrophages in the previous studies, with dioscin being a typical example.^495^ Dioscin is a natural compound with multiple functions including anti-inflammation and immunoregulation, which also means that dioscin is not specific for targeting HIF-1α expression in macrophages.^499^ In ulcerative colitis mice, dioscin can reduce M1 polarization and induce M2 polarization. Through the in vitro experiments, the inhibition of mTORC1/HIF-1α pathway is responsible for the reduced glycolysis observed under dioscin treatment.^495^ The PI3K/Akt pathway is a classic upstream pathway of mTORC1 and HIF-1α signaling.^500,501^ In T2DM mice treated with autologous blood transfusion, the upregulation of PI3K/Akt pathway in macrophages has been observed simultaneously with reduced levels of HIF-1α and glycolysis.^497^ In addition to macrophages, the glycolysis of effector T cells can also be reduced by inhibiting HIF-1α.^271^ An in vitro experiment has shown that costunolide treatment can induce HIF-1α degradation, leading to reduced glycolysis in Th17 cells and decreased Th17 cell differentiation.^271^ PKM2, a rate-limiting enzyme in glycolysis, is also an important target to control glycolysis in many diseases.^411,502,503^ The PKM2 inhibitor can alleviate microglial dysfunction of mice with Alzheimer’s disease by disrupting the upregulation of glycolysis.^473^ PKM2 is downstream of the PI3K/Akt pathway, indicating that inhibiting PKM2 may be one mechanism leading to reduced glycolysis through suppression of HIF-1α.^497^ 6-phosphofructo-2-kinase/fructose-2,6-bisphosphatase 3 (PFKFB3) is another downstream target of the PI3K/Akt pathway that induces glycolysis. Specific inhibitors of PFKFB3 have been used to reduce glycolysis and activity of inflammatory cells.^284,504,505^ As we mentioned before, GLUT1 also mediate glycolysis and can be regulated by the PI3K/Akt pathway. When exploring treatment options for blocking the glycolysis to inhibit M1 polarization in cardiac ischemia and reperfusion injury, researchers have found that sDR5-Fc can reduce GLUT1.^506^ In acute lung injury, phloretin has similar effects.^507^ With the downregulation of glycolysis in immune cells, the upregulation of AMP-activated protein kinase (AMPK) has been observed.^294,508,509^ AMPK is a guardian of mitochondrial homeostasis, and also controls lipid and glucose metabolism.^510^ Metformin, the first-line drug treatment for T2DM, has been found the capability to activate AMPK.^511^ Researchers have initiated an observational study to validate the capability of metformin in reversing the phenotypes of CD4^+^ T cells in obese children (NCT03960333). They believe that CD4^+^ T cells in obese children skew towards effector T cells rather than Treg cells due to upregulated mTOR-driven glycolysis and downregulated AMPK-driven OXPHOS. Unfortunately, their recently published results have not reported the role of metformin which was mentioned in their registration.^512^ Adiponectin can also inhibit glycolysis and the differentiation of Th17 cells by activating AMPK in obese mice.^509^ 2-deoxyglucose (2-DG) is a well-known glycolytic inhibitor.^513,514^ In RA rats, 2-DG can promote M2 macrophage polarization through activating AMPK, exerting anti-arthritic effects.^294^ In treating acute lung injury, 2-DG has shown the ability to reduce NLRP3 inflammasome.^487,515^ Adiponectin can inhibit the glycolysis of CD4^+^ T cells through activating AMPK, thereby reducing Th17 cell differentiation.^509^ The upregulated glycolysis of CD4^+^ T cells upon obesity can be induced by the increased FAO, promoting the activation of T cells and resulting in proinflammation, which also provides a viewpoint to target glycolysis.^334^ Carnitine palmitoyltransferase 1 (CPT-1) is a rate-limiting enzyme in FAO. CPT-1a is an important subtype of CPT-1 that stabilizes Goliath, a mitochondrial E3 ubiquitin ligase.^334^ Researchers have used a specific Goliath inhibitor, DC-Gonib32, to destroy the upregulated glycolysis of CD4^+^ T cells in obese mice.^334^

To treat cancer, inhibiting glycolysis of immune cells have also been studied. However, because cancer is dominated by an immunosuppressive environment, inhibiting glycolysis of immune cells in cancer besides immune cell-derived tumors should be discussed separately. JHU083, a glutamine antagonist, can simultaneously inhibit glycolysis in tumor cells and effector CD8^+^ T cells.^516^ However, when glycolysis is reduced, OXPHOS is upregulated in the CD8^+^ T cells, resulting in activating anti-tumor immune responses. This mechanism is related to TCA intermediate replenishment, which has been fully introduced.^516^ The function of L-arginine is similar to glutamine antagonists. L-arginine can induce a shift from glycolysis to OXPHOS in effector T cells, enhancing their anti-tumor immune effects.^102^ The researchers have conducted a randomized controlled trial to evaluate the efficacy of oral arginine in immune function improvement in patients with glioblastoma multiforme (NCT02017249), but the results have not been published. Hexokinase (HK) 2 is an important rate-limiting enzyme in glycolysis and has been identified as a potential therapeutic target for cancer.^517^ HK inhibitor can suppress glycolysis to block the PD-L1 expression, resulting in CD8^+^ T cell activation.^518^ In summary, contrary to suppressing inflammation, glycolysis inhibitors have shown the anti-tumor immune effects by enhancing OXPHOS, possibly because existing glycolysis inhibitors are unable to specifically target the glycometabolism of immune cells.

Upregulating glycolysis can enhance anti-tumor immune effects of immune cells including macrophages, effector T cells and dendritic cells.^175,519–522^ Vismodegib, a small molecule that can inhibit the hedgehog pathway, has been approved for the treatment of advanced basal-cell carcinoma.^523^ Later, in a breast cancer mouse model, vismodegib has been found to have the capability to shift the metabolism of M2 macrophages from FAO to glycolysis, thus alleviating immunosuppression.^520^ However, this effect has not been validated in human studies.^520^ The hedgehog pathway may participate in changing the metabolic patterns of immune cells.

### Targeting OXPHOS of immune cells to treat diseases

Also due to the non-specific targeting, to treat cancer, both downregulation and upregulation of OXPHOS have been taken into consideration, with downregulating OXPHOS being the less studied one.

Promising targets for cancer have been provided by existing studies fousing on OXPHOS downregulation of immune cells. FABP5 is a member of FABPs, a protein family known by its ability to promote cellular lipid uptake and intracellular transport, is expressed in T cells.^96^ Inhibiting FABP5 can cause mitochondrial changes and reduce OXPHOS in Treg cells, thereby impairing the suppressive activity of Treg cells due to the release of IL-10 induced by FABP5.^194^ IACS-010759 is a OXPHOS inhibitor developed for treating leukemia.^524^ IACS-010759 prefers to kill cells with Myc activity in the context of lymphoma.^525^ Myc can regulate metabolic reprogramming in cancer by upregulating glycolysis, and is also involved in mitochondrial diseases.^526^ However, why the OXPHOS inhibitor can specifically target cells with overexpressed Myc is unclear.^526^ In addition, in cancers with neoplastic lymphocytes, researchers also inhibit glycolysis as a treatment approach, similar to the downregulation of glycolysis by suppressing Myc function.^527,528^ It is important to note that regulating the metabolism of neoplastic lymphocytes aims to kill immune cells rather than regulate immune functions.

Anti-inflammatory and anti-tumor effects can be provided by upregulating OXPHOS to treat inflammatory diseases and cancer. The upregulation of OXPHOS is always accompanied by the downregulation of glycolysis, as discussed in the previous sections.^529^ Upregulating OXPHOS also has anti-inflammatory effects. NX-13, a new gut-restricted drug targeting leucine rich repeat containing X1, can upregulate OXPHOS and reduce NF-κB activation of CD4^+^ T cells in vitro, resulting in the inhibition of differentiation.^282^ A phase 1b study has reported that NX-13 is generally safe in patients with ulcerative colitis.^530^ The study has also showed that rectal bleeding and stool frequency can be improved at week 2, and endoscopic response can be observed at week 4.^530^ OXPHOS of CD8^+^ T cells can be significantly upregulated by including 4-1BB domain in the chimeric antigen receptor architecture, helping researchers to improve cancer immunotherapy.^531^ Linoleic acid, a commonly consumed polyunsaturated fatty acid, exhibits anti-inflammatory and anti-tumor effects.^532,533^ Although excessive intake of linoleic acid can lead to the formation of oxidized linoleic acid metabolites, increasing the risks of many diseases, linoleic acid also helps us to understand immunometabolism in cancer.^534,535^ Linoleic acid is a positive regulator of CD8^+^ T cells, upregulating calcium signaling and mitochondrial energy metabolism in CD8^+^ T cells.^536^ In HCC, T cells lose in the competition for linoleic acid with tumor cells, leading to T cell dysfunction and HCC progression.^537^ This suggests a protective role of linoleic acid in promoting anti-tumor immune responses.^537^

### Targeting lipid metabolism of immune cells to treat diseases

Lipid metabolism of activated or proinflammatory immune cells is predominantly dominated by lipid synthesis rather than lipid catabolism. Researchers have attempted to modulate the lipid metabolism of immune cells to alter the phenotype and function of immune cells for the treatment of diseases.

FAO is the most investigated lipid metabolic pathway in immunometabolism. Regulating FAO in immune cells has been explored to treat multiple disease including cancer, autoimmune diseases, metabolic disease, atherosclerosis and lung inflammation.^57,315,334,465,466,495,519,520,538,539^ FAO of macrophages is the most studied. Downregulation of FAO can be accompanied by upregulation of glycolysis, resulting in alleviating immunosuppression caused by M2 macrophages, which is one of the effects of the anticancer drug vismodegib.^520^ RIPK3 is a newly identified lipid metabolism regulator, contributing to the development of fatty liver, liver fibrosis, and HCC, with the signaling not fully explored.^540,541^ Although the upregulation of RIPK3 appears to be non‐protective in patients, the downregulation of RIPK3 in M2 macrophages during HCC also contributes to tumorigenesis.^57^ The downregulation of RIPK3 in macrophages can activate PPAR, leading to the upregulation of FAO and M2 polarization. Therefore, researchers have used a RIPK3 activator to downregulate FAO and suppress the immunosuppressive activity of M2 macrophages.^57^ The inconsistent effects of RIPK3 indicate that specific targeting of metabolism in immune cells is essential. Downregulating FAO in T cells has also been studied. CPT-1, the key rate-limiting enzyme in FAO, can promote cells to adapt to the environment when energy demand changes.^542^ In mice with MS, CPT-1 inhibitor treatment can alleviate neurological inflammation by reducing overall FAO, including in T cells.^315^

In most cases, upregulation of FAO in macrophages is usually anti-inflammatory. Upregulated FAO induced by dioscin is accompanied by decreased glycolysis, which collectively enhances M2 macrophage polarization and reduces inflammation in ulcerative colitis mice.^495^ The mTORC2/PPARγ signaling pathway may be a potential mechanism for dioscin to promote FAO in macrophages.^495^ The upregulation of PPARα has been repeatedly observed when upregulating FAO in macrophages.^465,539^ PPAR is a nuclear receptor with capacities of anti-inflammation and metabolic regulation.^543^ PPARα activators, including the fibrates, are commonly used for lowering lipid levels.^544^ The anti-inflammatory effects of PPARα activators support the importance of studying immunometabolism. Angiotensin-converting enzyme of macrophages can increase cellular lipid uptake and FAO by upregulating PPARα in the context of atherosclerosis.^465^ In respiratory syncytial virus induced lung inflammation, Qingfei oral liquid can upregulate PPARα to increase FAO, leading to M2 polarization.^539^ The Akt activator SC-79 can reduce the anti-inflammatory effects of Qingfei oral liquid by increasing FAS, validating the possible association between PPARα and Akt signaling in immunometabolism.^539^ However, the upregulated FAO of immune cells in cancer sometimes exhibits proinflammatory effects.^538^ CD40, a member of TNF receptor superfamily, can be activated to promote T cell activation and tumor infiltration.^545^ CD40 activation can induce FAO and glutamine metabolism in macrophages, promoting M1 polarization to enhance anti-tumor activity, which provides a side effect of anti-CD40 treatments in cancer.^538^ CD40 activators provide an anti-tumor effect of macrophages by upregulating FAO, indicating that FAO is not completely negatively associated with pro-inflammation. This could be attributed to its potential crosstalk with other metabolic pathways, which needs further discussion in the future.

The inhibition of lipid synthesis has been used in vitro to treat cancer and rheumatic diseases. Balancing normal immune tolerance and abnormal immunosuppression in the TME caused by Treg cells is a challenging issue. Researchers have found that inhibiting SREBP signaling can inhibit lipid synthesis and the activity of PI3K in Treg cells to suppress immunosuppression, resulting in inhibiting tumor growth without causing autoimmune toxicity.^195^ In CD8^+^ T cells from RA patients, LDHA is overexpressed, and researchers have used the LDHA inhibitor FX11 to reduce lipogenesis of CD8^+^ T cells, leading CD8^+^ T cells to lose their proinflammatory capacities.^297^ The results from the two studies have shown that inhibiting lipid synthesis is possibly able to downregulate anti-inflammatory effects of Treg cells and proinflammatory effects of CD8^+^ T cells, which should be validated in further studies.

Studies have explored the ability of approved drugs in inflammatory diseases and cancer to regulate lipid metabolism in immune cells. Tofacitinib, a Janus kinase inhibitor, is used to treat RA, ulcerative colitis, psoriatic arthritis, and other diseases.^546–548^ In rabbits with chronic arthritis, tofacitinib can reduce lipid accumulation and increase lipid release of macrophages by inhibiting JAK/STAT signaling pathway, thereby reducing systemic and synovial inflammation.^549^ The mechanism by which orlistat causes apoptosis in B-cell chronic lymphocytic leukemia cells may involve the inhibition of lipidolysis in B cells.^198^ In summary, studies on immunometabolism can help researchers to understand the mechanisms of current drugs.

Lipid peroxidation is a special lipid metabolism occurring in pathological and physiological conditions, and can regulate several cellular processes through its metabolites.^550^ In acute myeloid leukemia, an in vitro experiment has shown that the chenodeoxycholic acid treatment can inhibit M2 macrophage polarization by inducing lipid peroxidation through the activation of the p38 MAPK signaling pathway, resulting in the inhibition of tumor cell proliferation.^551^ However, lipid peroxidation and p38 activation related to CD36 can impair the function of CD8^+^ T cells in the TME. The use of a p38 inhibitor can restore CD8^+^ T cell function by upregulating GSH peroxidase 4 and inhibiting lipid peroxidation.^192^ N-acetylcysteine, a GSH precursor, has been shown to reduce the level of lipid peroxidation biomarker 8-iso-prostaglandin F2α in SLE mice, and activated FoxP3 expression in CD4^+^ T cells has also been observed.^552,553^

### Other treatments targeting metabolism of immune cells to treat diseases

Although glycometabolism and lipid metabolism of immune cells are still not thoroughly studied, other metabolic pathways have also shown their possible roles in immunometabolism.

Possibly benefiting from numerous studies on cancer metabolism, glutamine is almost the most studied amino acid in the field of immunometabolism. Downregulating glutamine metabolism has been considered as a promising treatment for cancer for a long time, given the addiction of cancer cells to nutrients and hypoxic environments.^554–557^ The heterogeneity of glutamine metabolism in cancer promotes researchers to seek more evidence.^555^ The inhibitor of glutamine metabolism can reduce immunosuppressive cells and increase proinflammatory macrophages.^207^ Along with the enhanced anti-tumor immune effects of macrophages, the blocked glutamine metabolism also occurs in tumor cells, inhibiting tumor growth.^207,558^ In triple-negative breast cancer, inhibiting glutamine metabolism can increase the presence of activated CD8^+^ T cells within the tumor.^559^ Later, other researchers have used JPH203, a SLC7A5 inhibitor, to target glutamine metabolism, resulting in increased CD8^+^ T cell infiltration.^560^ In lung cancer, the SLC7A5 inhibitor DRP-104 can inhibit glutamine-dependent nucleotide synthesis, enhancing the functions of CD4^+^ and CD8^+^ T cells while reducing Treg cells.^561^ In lymphoma, asparaginase can target tumor cells with high demand for glutamine.^562^ Subsequently, SLC1A1 has been identified as the key regulator that upregulates cellular glutamine metabolism.^232^ Asparaginase treatment can inhibit glutamine metabolism upregulated by SLC1A1 and restore impaired T-cell immunity in lymphoma.^232^ In summary, inhibiting glutamine metabolism can both suppress tumor cell growth and enhance anti-tumor immunity, providing potential strategies for other cells with reduced immune activity, such as those affected by infection. However, downregulating glutamine metabolism in cancer may actually upregulate glutamine metabolism of T cells. A metabolic competition exists between tumor cells and T cells. The glutamine transporter inhibitor V-9302 can selectively block glutamine uptake in tumor cells and improve the anti-tumor effects of CD8^+^ T cells.^209^ In addition, upregulating glutamine metabolism through the CD40 activator can enhance the proinflammatory and anti-tumor effects of macrophages, indicating that upregulating glutamine metabolism of immune cells is more beneficial for anti-tumor effects rather than immunosuppressive effects.^538^

Other metabolites or metabolic pathways have been studied in the field of immunometabolism. The serine metabolism of immune cells is also associated with cancer treatment. The serine synthesis inhibitor treatment can enhance NK cell activation and improve the efficacy of anti-PD1 immunotherapy.^563^ The serine/glycine metabolites can be reduced by lycorine to eliminate B-cell acute lymphoblastic leukemia cells, with phosphoserine aminotransferase 1 as a possible target.^564^ Lactic acid is a natural energy metabolite which can suppress anti-tumor immunity.^565^ Lithium carbonate, a mood stabilizer, can promote the transport of lactic acid into mitochondria of CD8^+^ T cells to obtain energy, resulting in the inhibition of immunosuppression.^566^

### Potential clinical applications related to immunometabolism

Although few drugs which are developed based on immunometabolism have been approved for clinical use (Table 2), existing studies have identified potential clinical applications associated with immunometabolism.

Measuring the metabolic activity of immune cells provides a possible method to perform early diagnosis and prognosis prediction. There are many indicators of cellular metabolism, such as oxygen consumption rate, extracellular acidification rate, mitochondrial membrane potential and enzymatic activity.^567,568^ CD14^+^ monocytes isolated from the blood of RA patients and individuals at risk have shown higher levels of glycolytic enzymes, including HIF-1α, HK2, and PFKFB3, than those from healthy individuals.^569^ The proinflammatory and hypermetabolic phenotype of CD14^+^ monocytes occur before RA onset, providing a potential method for timely diagnosis and treatment of RA.^569^ In glomerulonephritis, including nephrotic syndrome, antineutrophil cytoplasmic antibody-associated vasculitis, and SLE nephritis, renal biopsies of patients have shown that enzymes of the PPP are associated with macrophage markers.^570^ Due to the association between activation of the PPP and reduced kidney function, directly measuring related indicators of peripheral blood monocytes might be a rapid diagnostic method before renal biopsy.^570^ Isolating peripheral blood monocytes has been widely used in experiments, and the diagnostic and prognostic value of peripheral blood monocytes has been discussed in many diseases.^571–574^ In immunometabolism, there is significant interest in exploring the diagnostic and prognostic value of peripheral blood monocytes. Single-cell applications are also helpful for studies on immunometabolism.^575^ Additionally, some researchers have combined metabolic indicators, including body mass index with inflammatory indicators, such as C-reactive protein levels, to predict antidepressant treatment outcomes. This approach has been also defined as immunometabolism in their study.^576^

Metabolic regulation can be taken into consideration in cellular therapies based on immune cells. Chimeric antigen receptor (CAR)-T cell therapy is a new and rapidly developing immunotherapy against cancer.^577^ The interactions between the TME and CAR-T cells critically influence CAR-T cell function.^577^ Improving anti-tumor activity of CAR-T cells is important. The respiratory capacity and FAO of CD8^+^ central memory T cells can be significantly enhanced by including the 4-1BB signaling domain in the CAR architecture.^531^ Adding domains that can enhance the metabolism of immune cells provides a promising method for designing future CAR-T cell therapies. Exogenous immune cells with anti-inflammatory phenotype have been considered as a possible immunotherapy for autoimmune diseases.^578^ The researchers have infused Treg cells intravenously to four patients with autoimmune hepatitis and measured the metabolism of the exogenous Treg cells.^578^ Treg infusion has not cause high-grade adverse effects and further research is needed for efficacy.^578^

The immune checkpoint inhibitor, which targets immune checkpoints suppressing the activity of immune cells, is an important breakthrough in cancer.^579^ However, resistance to immune checkpoint inhibitors remains common, and metabolic intervention has been considered a promising therapy to enhance anti-tumor immune responses triggered by immune checkpoint inhibitors.^580^ The association between immune checkpoint inhibitors and the metabolism of immune cells is an important topic in the field of immunometabolism. Before the emergence of immunometabolism, cancer metabolism mainly focused on the abnormal metabolism of tumor cells. The metabolic reprogramming of immune cells in cancer is also important and has been discussed in the previous sections.

## Conclusions and perspectives

In this review, we have summarized how metabolism regulates immune effects in health and diseases, a concept known as immunometabolism. Immune cells exhibit abilities to metabolize various nutrients with complex metabolic reprogramming during activation. The multiple metabolic pathways affect the differentiation, phenotypes, and functions of immune cells. Among immune cells, macrophages and T cells are the most studied in the field of immunometabolism. Glycolysis, OXPHOS, FAO are the most studied metabolic pathways in immunometabolism. Immune cells with proinflammatory and anti-inflammatory phenotypes exhibit different metabolic patterns. Upregulation or downregulation of metabolic pathways can change the phenotypes of immune cells, resulting in regulation of immune effects. In the context of diseases, with cancer as the most studied one, metabolic reprogramming occurs in immune cells, promoting proinflammation or immunosuppression. Targeting metabolic pathways provides an attractive for the treatment of multiple diseases.

To translate experimental results into clinical applications, challenges remain in confirming the essential role of immunometabolism in disease treatment. A variety of factors, including different metabolic pathways, different cells and different diseases collectively influence the role of immunometabolism in treating diseases, which can be summarized as heterogeneity.^555^

First, heterogeneity exists in metabolic pathways and the effects of regulating metabolic pathways. The same regulation of metabolic pathways can lead to different immune effects. Upregulation of OXPHOS can induce anti-inflammatory M2 macrophage polarization, but can also activate T cells with proinflammatory effects.^52,80^ Upregulation of FAO can induce both anti-inflammatory polarization and inflammasome activation of macrophages.^57,58^ Active glutamine metabolism is associated with both M2 polarization and proinflammatory T cell differentiation.^60–63,104^ How generic metabolic pathways regulate immune effects is not sufficiently understood. It is more difficult to elaborate on the heterogeneity of metabolic pathways between immune cells and other cells, and further among different immune cells. Targeting metabolism of immune cells specifically is still difficult. A limitation of our study is that we have not discussed the crosstalk of different metabolic pathways in immune cells due to the limited evidence. A classic study in immunometabolism has discussed the intersection of glutamine metabolism and the TCA cycle in macrophages, but just one study may not be sufficient.^60^ In addition, available studies have paid much attention on glycolysis, but OXPHOS might have better targetability.^52^ Furthermore, existing studies on immunometabolism still focused on cellular metabolism of immune cells. How to expand cellular metabolic processes to metabolic processes without confusion is a big challenge for future studies on immunometabolism.

Second, heterogeneity exists in different cells. While the immunometabolism of macrophages and T cells are the most studied, it is important to recognize that other immune cells also play significant roles in the field of immunometabolism. We have summarized some metabolic pathways of B cells, but how metabolism affects the functions of B cells in diseases is not fully described. The number of subtypes of T cells are significantly more than that of macrophages. Further subdividing subtypes of immune cells may pose difficulties in research, but it can also obtain more reliable results. We guess that the contradictory results regarding the metabolism of macrophages may be resulted from the oversimplified classification of subtypes. Using quantitative indicators to replace M1 and M2 subtypes might be feasible. Additionally, Immunometabolism of other immune cells, including NK cells and ILCs, remains unclear. Besides immune cells, other cells relevant to various diseases should also be taken into consideration, including tumor cells in cancer, synovial fibroblasts in RA, adipocytes in obesity, and hepatocytes in NAFLD. If targeting conserved metabolic pathways, upregulating metabolism of immune cells can be accompanied by activating the cells that can promote disease development. The association between immune cells and other cells in the field of immunometabolism deserves further study. While the precise isolation of immune cells and specific cell types may be somewhat challenging, it is important.

Third, heterogeneity exists in different diseases. In many fields, cancer is always the most extensively studied disease, but studies on other diseases in immunometabolism are also important. Immune cells in cancer are dominated by an immunosuppressive phenotype, different from many inflammatory diseases such as autoimmune diseases and metabolic diseases. The prominent metabolic reprogramming of tumor cells may confuse the studies on metabolic pathways in immune cells. In cancer, new drugs are rapidly developed, so preclinical studies can be appropriate at present. However, in other diseases, exploring how widely used drugs control disease progression by regulating the metabolism of immune cells may be more feasible. Data from humans is precious. Randomized controlled trials are difficult to conduct, and observational studies are also valuable for obtaining data from humans. Distinguishing altered metabolism in tissues and immune cells can also be challenging, but important for exploring specific targets in immune cells, especially in diseases characterized by significant metabolism changes including obesity and T2DM.

In conclusion, metabolic pathways and metabolic reprogramming influence the phenotypes and functions of immune cells, thereby promoting disease progression. Based on experimental results, targeting the metabolic regulation of immune cells is a promising therapy for multiple diseases. To translate these experimental results into clinical applications, obtaining data from humans and elucidating mechanisms of immunometabolism in various metabolic pathways, different cells, and different diseases is necessary.
